# Re-investigation of functional gastrointestinal disorders utilizing a machine learning approach

**DOI:** 10.1186/s12911-023-02270-9

**Published:** 2023-08-26

**Authors:** Elahe Mousavi, Ammar Hasanzadeh Keshteli, Mohammadreza Sehhati, Ahmad Vaez, Peyman Adibi

**Affiliations:** 1 Department of Bioelectrics and Biomedical Engineering, School of Advanced Technologies in Medicine, Isfahan University of Medical Sciences, Isfahan, Iran; 2 Department of Bioinformatics, School of Advanced Technologies in Medicine, Isfahan University of Medical Sciences, Hezar Jerib Street, Po Box 8174673461, Isfahan, Iran; 3 Integrative Functional Gastroenterology and Hepatology Research Center, Department of Internal Medicine, School of Medicine, Isfahan University of Medical Sciences, Isfahan, Iran

**Keywords:** Functional gastrointestinal disorders, Unsupervised machine learning, Cluster analysis, Rome criteria, FGID

## Abstract

**Background:**

Functional gastrointestinal disorders (FGIDs), as a group of syndromes with no identified structural or pathophysiological biomarkers, are currently classified by Rome criteria based on gastrointestinal symptoms (GI). However, the high overlap among FGIDs in patients makes treatment and identifying underlying mechanisms challenging. Furthermore, disregarding psychological factors in the current classification, despite their approved relationship with GI symptoms, underlines the necessity of more investigation into grouping FGID patients. We aimed to provide more homogenous and well-separated clusters based on both GI and psychological characteristics for patients with FGIDs using an unsupervised machine learning algorithm.

**Methods:**

Based on a cross-sectional study, 3765 (79%) patients with at least one FGID were included in the current study. In the first step, the clustering utilizing a machine learning algorithm was merely executed based on GI symptoms. In the second step, considering the previous step's results and focusing on the clusters with a diverse combination of GI symptoms, the clustering was re-conducted based on both GI symptoms and psychological factors.

**Results:**

The first phase clustering of all participants based on GI symptoms resulted in the formation of pure and non-pure clusters. Pure clusters exactly illustrated the properties of most pure Rome syndromes. Re-clustering the members of the non-pure clusters based on GI and psychological factors (i.e., the second clustering step) resulted in eight new clusters, indicating the dominance of multiple factors but well-discriminated from other clusters. The results of the second step especially highlight the impact of psychological factors in grouping FGIDs.

**Conclusions:**

In the current study, the existence of Rome disorders, which were previously defined by expert opinion-based consensus, was approved, and, eight new clusters with multiple dominant symptoms based on GI and psychological factors were also introduced. The more homogeneous clusters of patients could lead to the design of more precise clinical experiments and further targeted patient care.

**Supplementary Information:**

The online version contains supplementary material available at 10.1186/s12911-023-02270-9.

## Introduction

A spectrum of chronic gastrointestinal (GI) symptoms, with no well-known structural or physiological abnormality, manifested a group of disorders named functional gastrointestinal disorders (FGIDs). Despite various findings on the correlated factors with specific FGIDs, no consistent biomarker has yet been introduced as the underlying cause of these conditions.

Regardless of sex, age, or ethnicity, all individuals are susceptible to these disorders, which can also cause serious negative effects on the quality of life in addition to enforcing an economic burden on healthcare systems [1, 2].

The Rome Foundation has established consensus-based criteria (called the Rome criteria) for the diagnosis and categorization of FGIDs [3]. According to the Rome criteria, FGIDs can be characterized based on a single symptom, such as functional dysphagia (FDG), or a mix of symptoms, such as irritable bowel syndrome (IBS) [4]. Although FGIDs involve different regions of the digestive tract, it is observed that diverse symptoms may occur in the same patient, frequently [5, 6].

Furthermore, due to the emergence of a continuum in FGIDs and the abundance of scientific evidence on their overlaps, it is challenging to distinguish between them with confidence [7–10]. Various studies focused on the overlaps of two or three specific FGIDs to investigate if new syndrome may be intruded due to the overlaps, but considering the whole spectrum of FGIDs requires a more elaborate and comprehensive framework [11–16].

In addition to GI symptoms, it is generally accepted that individuals with FGIDs also have psychosocial symptoms such as stress, anxiety, and depression, and researchers acknowledged the term "gut-brain axis" to summarize the bidirectional communication pathways between the gut and the brain [17–19]. According to a systematic review and meta-analysis focusing on IBS as one of the most prevalent FGIDs, combining GI symptoms with biomarkers and/or psychological affect indicators (such as anxiety, depression, or somatization) performs better in identifying IBS in general [20, 21]. However, despite the relationship between GI symptoms and extra-intestinal manifestations, such as psychological factors, the latter have not been considered in the Rome classification.

As a hypothesis, it could be asked whether considering psychological factors alongside GI factors could tackle the multi-labeling problem of the Rome classification. To be able to consider a wider spectrum of features and with the aim of finding more homogeneous groups for patients, in the current study, we used an unsupervised learning method to re-cluster FGID patients. Identifying more homogeneous and well-separated groups of patients could provide more focused clinical trials to better understand the underlying mechanisms.

## Material and methods

### The study population

This research was carried out as part of a cross‐sectional study named Study on the Epidemiology of Psychological‐Alimentary Health and Nutrition (SEPAHAN) conducted in 2010 [22]. Utilizing a multistage random cluster sampling, various information from 4763 non-academic staff was acquired through self-administered questionnaires. Demographic, dietary data, and the Rome III questions were accumulated, as well as the General Health Questionnaire as a measure of current psychological distress [23], the Hospital Anxiety and Depression Scale [24], and NEO Five‐Factor Inventory to measure personality traits [25]. The acquired data of 3765 individuals who fulfilled the Rome III criteria for having at least one FGID were applied in the current study.

### Demographic and GI symptoms

To evaluate the frequency of GI symptoms a 4-point Likert scale questionnaire was utilized. These symptoms were related to common FGIDs including functional heartburn (FHB), functional chest pain (FCP), functional dysphagia (FDG), Globus (G), functional dyspepsia (FDP), belching disorder (B), nausea and vomiting (V), rumination syndrome (RS), irritable bowel syndrome (IBS), functional bloating (FB), functional constipation (FC), functional diarrhea (FDI), functional fecal incontinence (FFI), and functional defecation (FDF) in Adults.

### Psychological factors

The NEO Five-Factor Inventory (NEO-FFI) was used to examine people's personality scores [26]. The NEO-FFI consists of sixty self-descriptive statements intended to evaluate personality traits based on five factors: I) Neuroticism: proclivity for negative effects and instability; II) Extraversion: sociability and energetic activity; III) Openness: reflects an individual's interest in new people, ideas, and intellectual qualities; IV) Agreeableness: a tendency to amiability; V) Conscientiousness: attributes including punctuality, goal-orientation, and dependability. These factors cover the main dimensions of personality, each with 12 items on the 5-point Likert scale.

The 14-item Hospital Anxiety Depression Scale (HADS) was used to assess anxiety and depression severity [24, 27]. Each item is rated on a 4-point scale, with the anxiety and depression subscales each receiving a maximum of twenty-one. A score of 8–21 on either subscale indicates psychological issues, whereas a score of 0–7 is considered normal.

The general health questionnaire (GHQ)-12 was used to assess psychological distress using a validated Persian version of the questionnaire [28]. The GHQ-12 has 12 questions with a 4-point rating scale and uses a bimodal scoring system (0011) to determine distress level. The probable range of scores is 0–12, with higher scores indicating more psychological distress.

### Statistical methods

Considering the current limitations of the Rome classification for FGIDs, particularly the presence of patients with multiple FGIDs, to identify more homogeneous and pure groups of patients in the population, we proposed to redefine syndromes using an automatic and unsupervised learning (clustering) method based on a number of GI symptoms and psychological factors simultaneously.

Most clustering algorithms are based on determining the distances or similarities between samples. Due to the attributes’ different natures, defining a unified distance to improve the clustering performance for a mixed data set composed of nominal, ordinal, and numerical attributes is very challenging. The Generalized Unified Distance Metric (GUDMM), which was created for mixed-type data, is a distance measure that takes into account the relationship between variables as well as the distributional information for different types of variables. In this regard, entropy and Jensen Shannon divergence concepts were used to exploit the inter-attribute information of categorical-categorical and categorical-numerical attributes, respectively. Furthermore, using a modified version of Mahalanobis distance, the intra- and inter-attribute information of numerical attributes was employed. Through a unified framework defined based on mutual information, the attributes’ contributions to distance measurement were controlled. This distance, in conjunction with the spectral clustering approach (GUDMM-S) [29], has offered a comprehensive platform for clustering mixed-type datasets, which is consistent with the input variables of the current investigation, namely ordinal GI measures and continuous psychological scores.

We implemented GUDMM-S in two phases. The clustering method was initially applied to only thirty GI symptoms. One of the main objectives of this study was to introduce new clusters for people who reported multiple GI symptoms and to assess the role of psychological factors in determining these groups. Therefore, we separated the resultant clusters of the first step into two groups: pure clusters and non-pure clusters. This separation was performed by an investigation of the cluster profile, visually and also compactness score of each cluster. The dominance of one variable was clearly visible in the radar plot of GI symptoms for pure clusters, whereas in non-pure clusters, the dominance of multiple GI variables was manifested. In the second step, further clustering for the members of non-pure clusters was performed to find more homogeneous clusters from the perspective of both GI symptoms and psychological factors.

The number of clusters in both steps, were determined using the internal clustering evaluation index, S-Dbw [30]. This evaluation index consisting of two components: intra-cluster compactness and inter-cluster separation. In order to evaluate the compactness of the dataset, the average variances of clusters are calculated, reflecting the extent of scattering within clusters. A lower value for this component signifies the presence of compact clusters. On the other hand, achieving a high degree of separation between clusters necessitates a significant reduction in density among clusters compared to the density within each individual cluster. Consequently, S-Dbw incorporates the concept of inter-cluster separation by examining the average density in the inter-cluster region relative to the density within the clusters themselves. A smaller value for this component also indicates well-separated clusters. By summing up these two terms, the S-Dbw index can be obtained, with the minimum value indicating the optimal number of clusters.

To investigate the stability of the clustering results achieved by GUDMM-S, we examined the results by subsampling rate of 0.02, 0.04, 0.06, 0.08, and 0.1. In each iteration of subsampling, 10 repetitions of clustering were performed for a range of number of clusters, from 2 to 15, and the clustering accuracy (CA) using Hungarian method were calculated [31, 32]. The average of CA over subsampling rates and repetitions has been calculated and the stability of results has been confirmed by achieving the CA more than 85% for identified number of clusters.

## Results

### Descriptive data of the study population

In this study, we analyzed data of 3765 patients who met the Rome III criteria for at least one FGID. Their mean age was 36.6 ± 8.1 years and 55.8% were female. In this population, the majority of participants had experienced more than one FGID (74.98%). Table 1 provides more information on the distribution of different FGIDs in this population.

**Table 1:** Distribution of study population based on Rome III classification

Disorder	Overlapped Samples		Pure samples		Disorder	Overlapped Samples		Pure Samples	
	Number (%)		Number (%)			Number (%)		Number (%)	
**FHB**	1000	(26.6)	78	(2.1)	**FC**	618	(16.4)	88	(2.3)
**FCP**	732	(19.4)	85	(2.3)	**FB**	725	(19.3)	180	(4.8)
**G**	269	(7.1)	18	(.5)	**FDI**	12	(0.3)	3	(0.1)
**FDG**	290	(7.7)	19	(0.5)	**FFI**	140	(3.7)	7	(0.2)
**FDP**	672	(17.8)	28	(0.7)	**FDF**	484	(12.9)	0	(0)
**PF**	521	(14)	26	(0.7)	**IBS**	948	(25.2)	40	(1.1)
**EP**	354	(9.4)	7	(0.2)	**IBS-C**	251	(8.9)	2	(0.1)
**B**	2137	(56.8)	351	(9.3)	**IBS-D**	151	(6)	5	(0)
**V**	938	(24.9)	45	(1.2)	**IBS-M**	126	(4.9)	3	(0.1)
**RS**	12	(0.3)	0	(0)	**IBS-U**	198	(6.9)	12	(0.1)

Overlapped samples refer to the population with more than one FGID, and pure samples contain individuals with only one FGID

*FHB* indicates functional heartburn, *FCP* Functional chest pain, *G* Globus, *FDG* Functional dysphagia, *FDP* Functional dyspepsia, *PF* Postprandial fullness, *ES* Epigastric pain syndrome, *B* Belching, *V* Vomiting, *RS* Rumination syndrome, *FC* Functional constipation, *FB* Functional bloating, *FDI* Functional diarrhea, *FFI* Functional fecal incontinence, *FDF* Functional defecation disorder, *IBS* Irritable bowel syndrome, *IBS-C* Constipation predominant IBS, *IBS-D* Diarrhea predominant IBS, *IBS-M* Mixed-type IBS, and *IBS-U* Unclassified IBS

### First step clustering analysis: clustering based on GI symptoms

Given that the Rome classification was based on expert consensus and was solely focused on GI symptoms, we attempted to examine the population in the first stage using the same core variables as Rome but using a machine learning technique. The majority of the resultant clusters at this stage matched the Rome-defined symptoms. In other words, this phase confirmed the capability of the GUDMM-S method for clustering this data.

Using z-values for each of the included variables, we created a radar plot for each cluster. We estimated them by adjusting each variable's cluster mean to the population mean normalized by the standard deviation. In addition to the radar plots, statistical tests were used to compare the variables in each cluster against the rest of the samples, which confirm the dominance of a symptom in the cluster. Stacked bars of GI symptoms and box plot of psychological factors to demonstrate the distributions of symptoms in each cluster were also provided, which are useful for the interpretation of variables in clusters, particularly for those within the average population's range. We next compared and defined the variables qualitatively (i.e., high, moderate, and low) in clusters using the statistical tests and graphs stated above. The variables with values of more than 0.5σ from the population mean or having *p*-values < 0.001 and an effect size of more than 0.3 were used to define a variable as “high” in a cluster. In the same way, for values of variables placed in the central circle, the variable’s level was considered low, and the range in this between was defined as *moderate*.

**Fig. 1:**
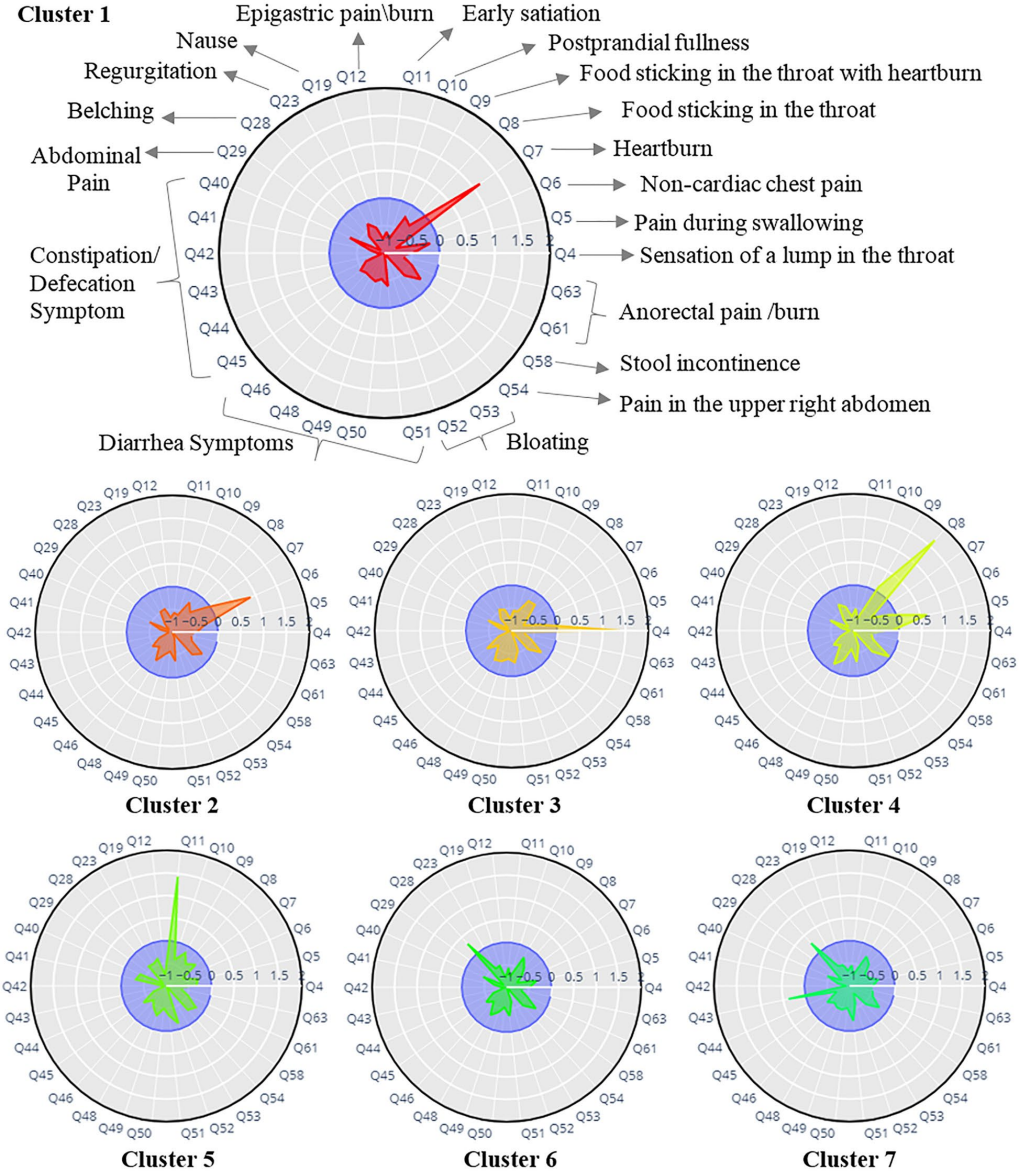
Radar plots of clusters identified by the GUDMM-S method based on GI symptoms in the first step. Clusters 1–13 indicate specific characteristics, while clusters 14–16 represent non-pure clusters. Clusters 1–13 are almost indication of one of the Rome III syndromes as follows: Cluster1 ~ Functional heartburn, Cluster 2 ~ Functional chest pain, Cluster 3 ~ globus, Cluster 4 ~ Functional dysphagia, Cluster 5 ~ Postprandial fullness, and Clusters 6, 7 ~ Belching

**Fig. 2:**
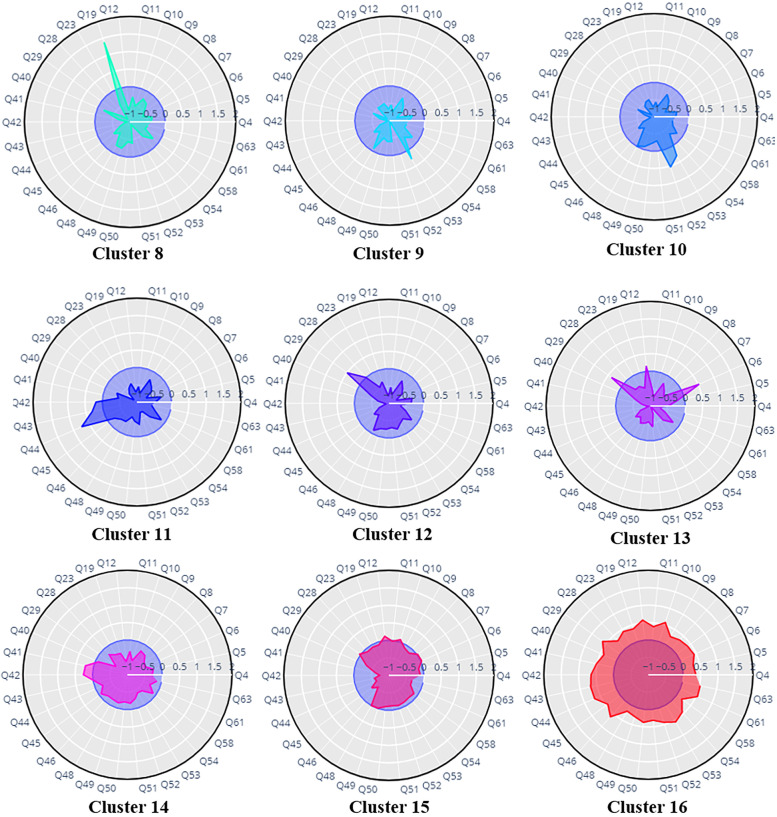
(Continued). Cluster8 ~ Nausea, Cluster 9, 10 ~ Functional bloating, Cluster 11 ~ Functional constipation, Cluster 12 ~ Abdominal pain, and Clusters 13 ~ Pain in epigastric, chest, and abdomen

To determine the number of clusters, we used the S-Dbw internal clustering evaluation index [30]. The minimum values of this index with respect to the number of clusters determine the proper number of clusters. The graph of the S-Dbw index is indicated in Supplementary Fig. 1. Sixteen clusters were recognized based on GI symptoms using GUDMM-S methods. According to the symptom profile presented in Figs. 1 and 2, all clusters except the last three clusters represent one of the FGIDs introduced by Rome III. Accordingly, the last three clusters were identified as non-pure clusters and the rest as pure clusters.

FHB is defined by the presence of heartburn symptoms, and cluster 1 is also characterized by a high frequency of heartburn. Cluster 2, by indicating the dominance of non-cardiac chest pain, is a representation of FCP. The only very high-frequency variable in cluster 3 is the sensation of a lump in the throat, which defines the Globus disorder. Considering the significant features of cluster 4, i.e., pain during swallowing and food sticking in the throat, cluster 4 is the equivalent disorder of FDG. The presence of early satiation in cluster 5 indicates the presence of postprandial fullness, which is indicative of FDP. Belching is the most common trait in two clusters, 6 and 7. Considering the high prevalence (> 90%) of nausea in cluster 8, patients in this cluster can be labeled as having “functional vomiting”, “cyclic vomiting”, or “chronic vomiting”.

The dominant feature of cluster 9 is bloating, which can be indicative of FB according to the Rome III criteria. Furthermore, two dominant variables in cluster 10 are stomach growling and bloating, which also indicate the presence of FB. Three questions about constipation-related symptoms, including straining during defecation, sensation of incomplete evacuation, and sensation of anorectal obstruction, are the main characteristic symptoms of cluster 11. Thus, cluster 11 is an indication of FC disorder. Cluster 12 is characterized by the dominance of abdominal pain, and cluster 13 indicates a higher frequency of heartburn and abdominal pain. Clusters 14, 15, and 16, encompass a wide variety of symptoms and overlapped FGIDs. The proportion of FGIDs in identified clusters is represented in Supplementary Table 1, in detail.

### Second step clustering analysis: clustering based on GI and psychological factors

Considering the profile of participants in clusters 14, 15, and 16, and the prominence of more than one or two symptoms, the overlaps of different FGIDs can be identified in these clusters.

**Fig. 3:**
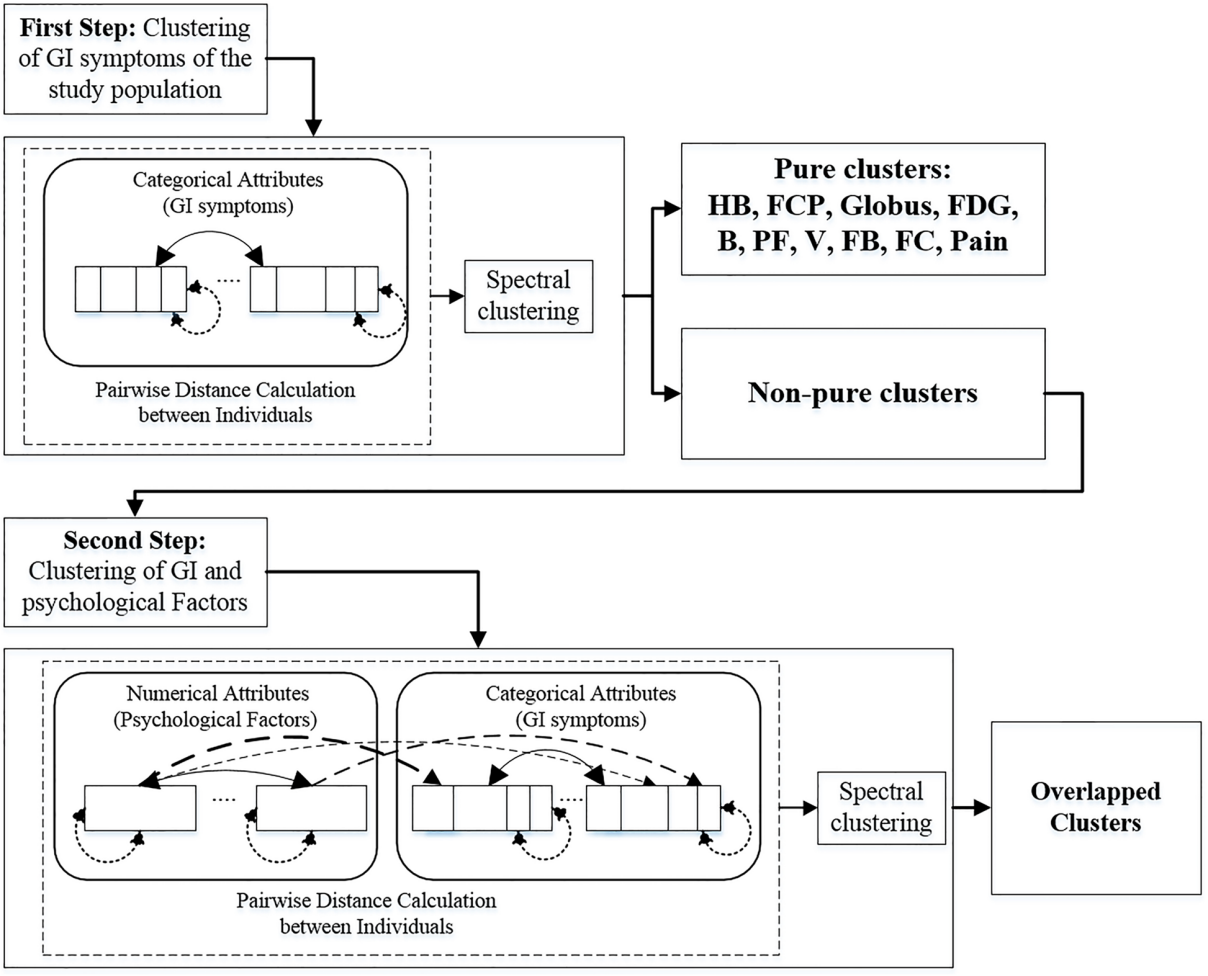
Flowchart of the proposed procedure for clustering of FGIDs

**Fig. 4:**
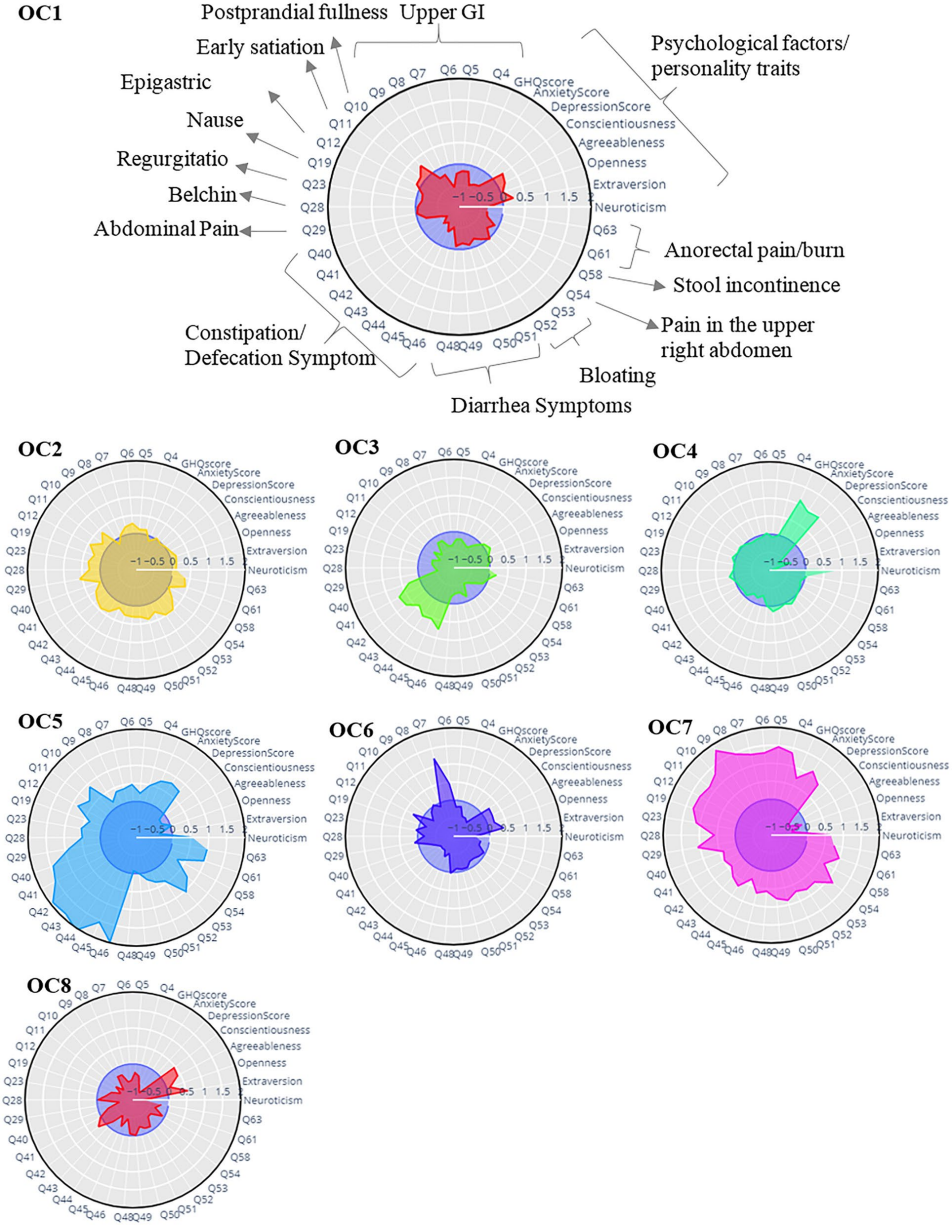
Radar plots of overlapped clusters (OCs) identified in the second step

To evaluate the effectiveness of psychological factors in combination with GI symptoms in finding more homogenous groups of patients, all samples from clusters 14, 15, and 16, including 2990 individuals, were re-clustered using 30 GI symptoms as well as anxiety and depression scores, the five personality dimensions of the NEO questionnaire (i.e., neuroticism, extraversions, openness, agreeableness, and conscientiousness), and GHQ12. Based on the S-Dbw internal validation index, eight clusters were selected as the suggested number of overlapped clusters (OCs) (Supplementary Fig. 2). A summary flowchart of the proposed procedure and the profile of the OCs have been indicated in Figs. 3 and 4, respectively.

OC1 is associated with moderate postprandial fullness, bloating, epigastric and abdominal pain, and a low psychological burden. These symptoms indicated the co-occurrence of FDP and IBS in one cluster.

Considering both the radar plot of OC2 indicated in Fig. 4 and the effect sizes reported in Supplementary Table 2, the predominance of lower GI symptoms and abdominal pain, as well as the moderate presence of upper GI symptoms and psychological burden, are the main characteristics of individuals in OC2.

The major representatives of participants in OC3 were constipation symptoms, as well as mild upper GI symptoms and a low psychological burden. The main difference of this OC with pure FC cluster identified in the first clustering step refers to the co-occurrence of rectal pain/burn and constipation symptoms in latter cluster.

OC4 was characterized by the moderate upper and lower GI symptoms with high psychological burden. In terms of Rome classification, FHB, FCP, G, FDG, FDP, V, FC, and IBS have been observed in OC4.

OC5 with high lower (except diarrhea) and upper GI symptoms, and high psychological burden and OC7 with high lower (except constipation symptoms) and upper GI symptoms and high psychological burden are two clusters with highly overlapped symptoms.

OC6 has a high prevalence of heartburn, as well as significantly below average constipation symptoms and psychological burden. Further to the presence of chest pain and heartburn, the stacked bar of OC6 has represented the prevalence of belching, abdominal pain, and bloating, which have been indicated in the radar plot in the average range of the population (indicated in the Supplementary Figs. 3 and 4). FHB accounts for about 96% of the OC6 in terms of Rome classification, with IBS accounting for a smaller percentage.

Considering the profile of clusters indicated in Fig. 4 and the results of the statistical tests indicated in supplementary Table 2, OC8 is a cluster in which its members mostly suffer from two symptoms of bloating and belching.

The description of eight OCs is summarized in Table 2, and box plots of psychological factors are also demonstrated in Supplementary Figs. 5, to provide a complete description of OCs.

**Table 2:** Description of the eight overlapped clusters found for 2990 patient of the second step

	**Description**	**Number of samples (%)**	**Female (%)**	**Mean age (SD)**
OC1	Predominant postprandial fullness, average bloating, epigastric and abdominal pain, and low psychological burden	441(0.15)	245(0.56)	36.39(7.21)
OC2	Predominance of all lower GI and abdominal pain, average upper GI symptoms and psychological burden	577(0.19)	364(0.63)	36.41(7.51)
OC3	Predominant constipation, low other GI symptoms and average psychological burden	292(0.1)	206(0.71)	36.22(7.44)
OC4	Moderate GI symptoms and high psychological burden	533(0.18)	355(0.67)	35.45(6.79)
OC5	High overall GI symptom with (except diarrhea), and high psychological burden	260(0.09)	191(0.73)	36.83(7.72)
OC6	Predominant heartburn and chest pain, low constipation and psychological burden	250(0.08)	113(0.45)	37.4(7.53)
OC7	High overall GI symptom (except constipation) with high psychological burden	271(0.09)	172(0.63)	36.24(6.84)
OC8	Average Constipation, low other GI symptoms and low psychological factors	366(0.12)	168(0.46)	35.86(7.48)
*P* value			< .001	0.06

The results of the multiple comparison test and the post-hoc analysis of symptoms in eight identified OCs of the second step, based on Kruskal–Wallis followed by Conover and Holm correction, have been summarized in the supplementary Table 2 and supplementary Figs. 6–8. In addition to the MCT test of the input variables, to investigate the statistical difference of somatic symptoms in each cluster vs. the rest of the population, the results of the Man-Whitney U test were also reported in supplementary Table 2. While the results of the Kruskal–Wallis test indicate the significantly different values of GI and psychological factors in eight OCs, the values of effect size for some variables are small, which declares their lower importance in discrimination of clusters. Openness, belching, fecal incontinence, and high blood pressure had the lowest values of effect size. The values of effect size for somatic symptoms in comparison to other clusters are especially higher in OC5 and OC7, where most of the other GI and psychological factors are also dominant. In Supplementary Table 3, the proportion of FGIDs in eight identified OCs is indicated.

## Discussion

In the current study, with the aim of improving the results of the Rome criteria definition for grouping the patients, we re-clustered the patients with at least one FGID using a machine learning method. While previous studies on suggesting new groups for FGID samples failed to confirm the existence of Rome subgroups and proposed some broad definition for individuals based on GI symptoms, by utilizing a proper unsupervised learning method, we demonstrated the presence of discriminant groups of individuals based on GI symptoms that mainly characterized by the prominence of one or two symptoms and correspond to most of the Rome subgroups, in addition to eight OCs who had multiple dominant symptoms based on a combination of GI and psychological symptoms.

By considering the clustering procedure of first step followed by the second step, splitting the non-pure clusters of 14, 15, and 16 is thoroughly obvious (see Supplementary Fig. 9). Our experiments indicated that even by increasing the number of clusters to 21 in the first step, the last three clusters were still almost stable, and the separation of individuals led to some other small-sized clusters, which indicated the overlap of some GI symptoms. However, when the psychological components were added in the second stage, these clusters were separated into more homogeneous clusters. Cluster 14, which showed the dominance of constipation symptoms in addition to the existence of other symptoms, in the second step, was broken into OC3, OC4, and OC8. Both OC3 and OC8 represent constipation, but at different levels of psychological burden. Cluster 15 is another non-pure cluster that shows different GI symptoms except constipation. The addition of psychological factors separated this cluster into OC1, OC4, and OC6, which indicate the different psychological burdens in addition to various levels of GI factors. The fraction of Cluster 16 resulted in the spread of its samples to almost eight new clusters.

While all identified OCs indicated multiple noticeable GI symptoms, OCs 1, 3, 6, and 8 demonstrated high/moderate levels for some conceptually related GI variables and low levels for other variables. The psychological burden showed low levels in these 4 clusters. The pure clusters of step 1, also indicated a low psychological burden. However, the high psychological burden was observed in two OCs 5 and 7, where the levels of GI symptoms were high in multiple upper and lower GI variables. OCs 2 and 4, with a moderate level for multiple GI symptoms, also indicated a moderate and high psychological burden, respectively. In other words, in our clusters, the co-existence of *multiple moderate/high level-upper and lower GI symptoms* coincided *with moderate/high psychological factors*, whereas clusters with *limited dominant GI symptoms* indicated *low levels of psychological burden*. Thus, it seems that designing clinical trials based on the inclusion of individuals with multiple GI and psychiatric problems with moderate/high levels of factors instead of focusing on one or two specific FGIDs with any level could provide more information on the bidirectional nature of the gut-brain axis.

Choung et al. [33], investigated the overlap of GERD and FDP and discovered the impact of excessive somatization, particularly insomnia and proton pump inhibitor use, in dyspepsia-GERD overlap. Considering the profile of symptoms in the overlapped group, it may represent a separate syndrome, according to this study. Choi et al. [13], evaluated the clinical and demographic features of FDP, IBS, and IBS-FDP overlap. They found that patients with IBS-FDP overlap had more severe symptoms (such as nausea, vomiting, bloating, hard or lumpy stools, straining, and a feeling of incomplete bowel movement) and higher depression scores compared with non-overlapped patients. In our experiments, C5 is equivalent to pure postprandial fullness (PF) which its member has low psychologic burden, and OC2, OC5, and OC7 which included the most individuals with both IBS and FDP. These OCs indicated the average and high values of psychological burden.

Besides the existence of various studies on the overlapped FGIDs based on the co-occurrence of two or three FGIDs [34–36], there are only a few studies that have been conducted on the analysis of whole FGIDs at the same time. Latent class analysis (LCA) is a learning algorithm that has been used to find similar groups of patients based on GI symptom levels, somatization scores, age, and gender [37]. Four groups characterized by asymptomatic, upper abdominal symptoms, lower abdominal symptoms, and mixed (upper and lower abdomen) symptoms are the reported clusters, which are very general definitions for the identified groups.

Another endeavor for the reinvestigation of FGID groups [38], by employing k-mean clustering and factor analysis, resulted in four groups: 1) an abundance of IBS-like symptoms; 2) meal-related discomfort, fullness, and bloating; 3) food regurgitation and diarrhea; and 4) painful symptoms. In that study, the variation that was covered by the nine factors was about 55%. The accumulated variance of the dominant factors based on only GI symptoms, in our experiments was less than 40%. Data projection based on a few dominant factors with low explained variance may omit a vast range of data variations and result in a failure to execute comprehensive clustering.

What has not been assessed in our study and other studies [39, 40], is the investigation of the onset of symptoms. The role of the brain-gut axis or the gut-brain axis in the formation of clusters with low or high psychological factors should be considered in future research. Given that the individuals first experienced GI symptoms or psychological symptoms may possibly better explain the reason for the existence of groups with similar GI and psychological characteristics but in different frequency and severity level.

Although the current study was conducted on a population-based dataset with the advantage of accessing the full spectrum of symptoms in the entire population and not being biased by health care seeking as in patient-based studies, possible cultural or genetic factors may affect the results due to the national nature of the study. Furthermore, because the research population existed prior to the publication of the Rome IV criteria, the patients were labeled using the Rome III criteria, and our inclusion criteria is based on Rome III. Despite the fact that our system was able to identify a single abdominal pain-related cluster in the first stage, IBS and epigastric pain syndrome were the only FGIDs that our algorithm could not identify as a single FGID (among the most common FGIDs).

## Conclusion

In the current study, by employing the concept of gut-brain interaction, a new grouping for FGID patients was created. It was demonstrated that learning-based systems are capable of the construction of patient clusters based on a broad variety of factors simultaneously by employing a database instead of expert-opinion-based grouping. Furthermore, the more homogenous clinically-defined clusters could also assist in future trial designs for finding the underlying mechanisms, more precisely.

### Supplementary Information

**Additional file 1. **

## Data Availability

The python implementation that support the findings of this study are available from the corresponding author upon reasonable request.

## Abbreviation

FGIDs: Functional gastrointestinal disorders
FHB: Functional heartburn
FCP: Functional chest pain
FDG: Functional dysphagia
G: Globus
FDP: Functional dyspepsia
B: Belching disorder
V: Nausea and vomiting
RS: Rumination syndrome
IBS: Irritable bowel syndrome
FB: Functional bloating
FC: Functional constipation
FDI: Functional diarrhea
FFI: Functional fecal incontinence
FDF: Functional defecation
NEO-FFI: NEO five-factor inventory
HADS: Hospital anxiety depression scale
GHQ: General health questionnaire
GUDMM: Generalized unified distance metric
MCT: Multiple comparison test
OCs: Overlapped clusters
LCA: Latent class analysis
FGIDs: Functional gastrointestinal disorders
FHB: Functional heartburn
