# Supplementary material for: Re-investigation of functional gastrointestinal disorders utilizing a machine learning approach
Source: BMC Med Inform Decis Mak. 2023 Aug 26;23:167. doi: 10.1186/s12911-023-02270-9 (PMC10463372; doi:10.1186/s12911-023-02270-9)
Supplement: Supplementary file 1 — Additional file 1. [file 12911_2023_2270_MOESM1_ESM.docx]

**Re-investigation of Functional Gastrointestinal Disorders**

**Utilizing a Machine Learning Approach**

**Supplementary Figures**


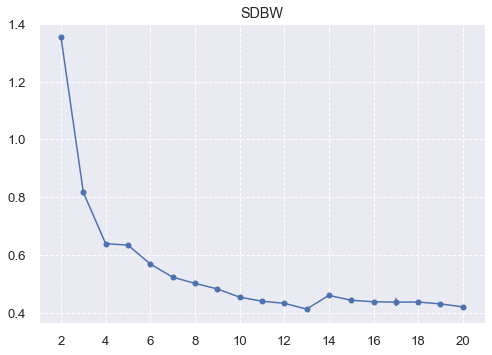


Supplementary Figure 1. S-Dbw index for determining the number of clusters.


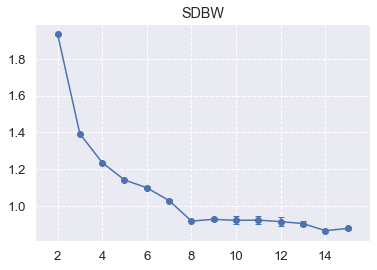


Supplementary Figure 2. S-Dbw index for determining the number of clusters in the second step.


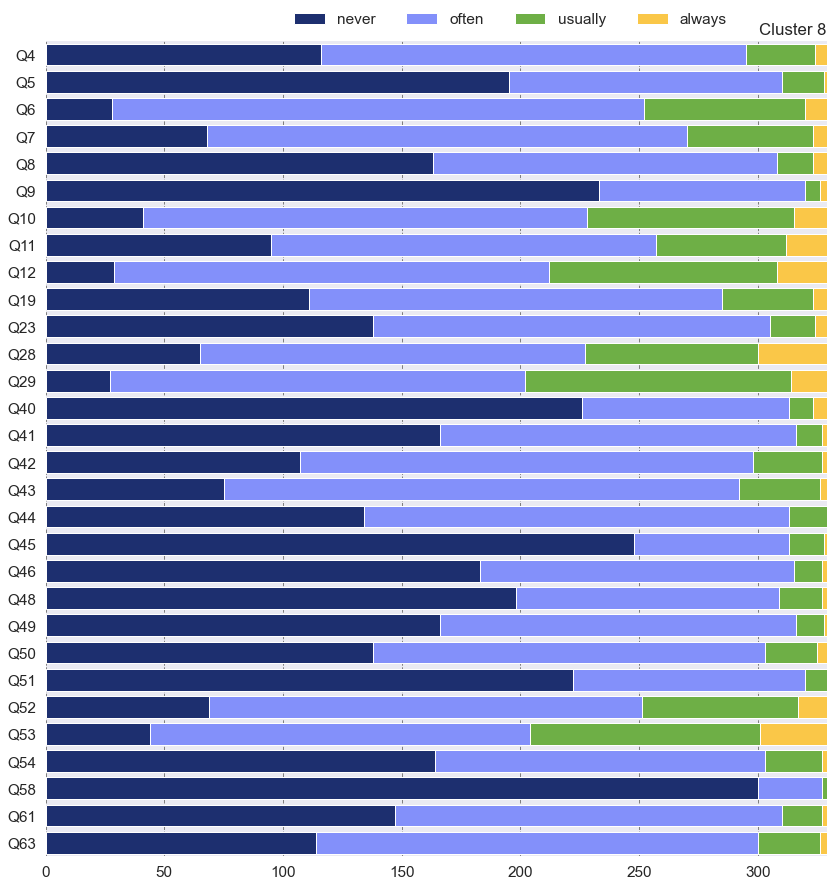

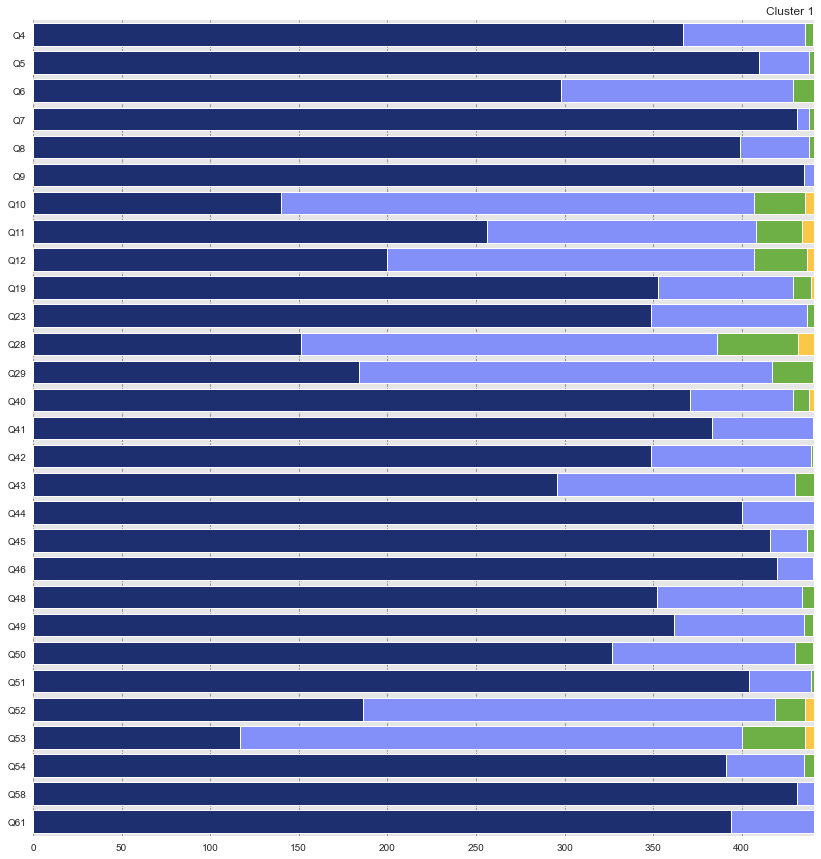

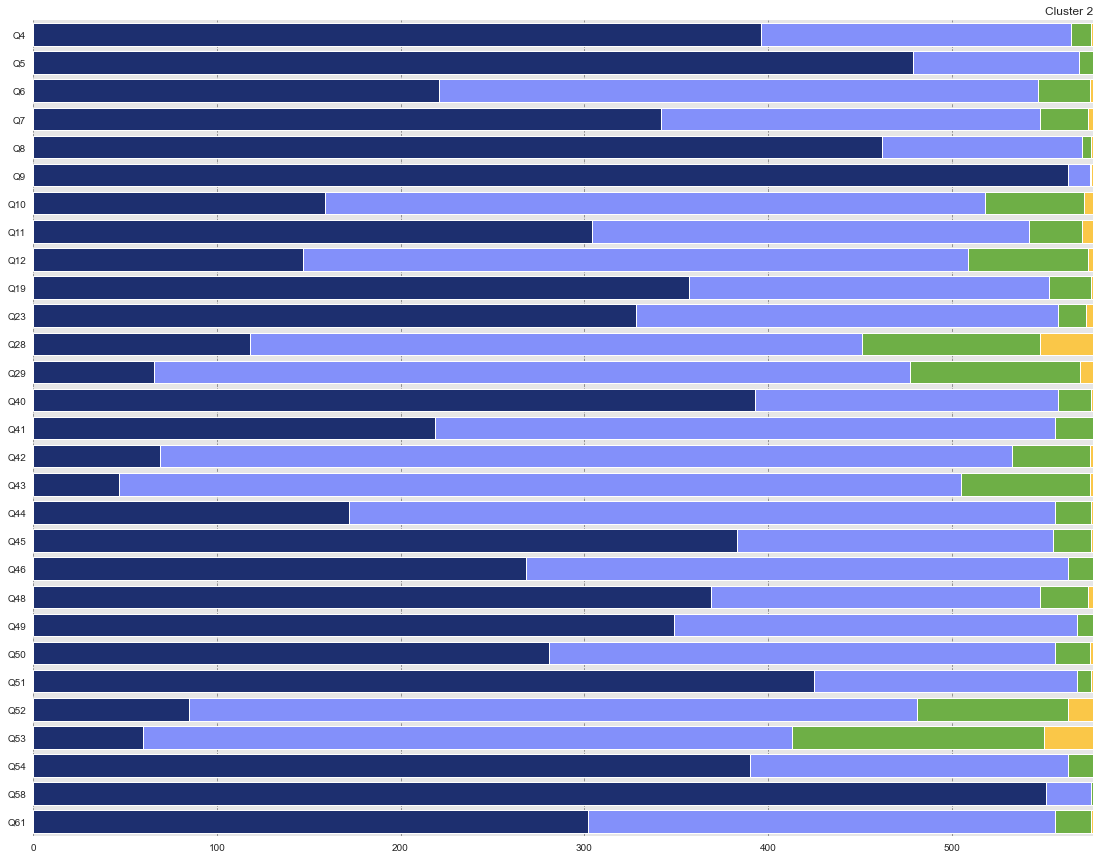

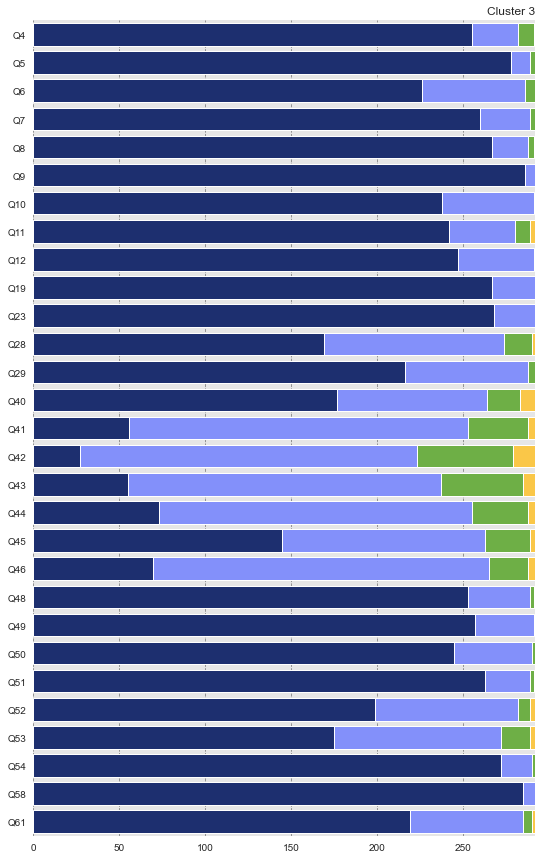

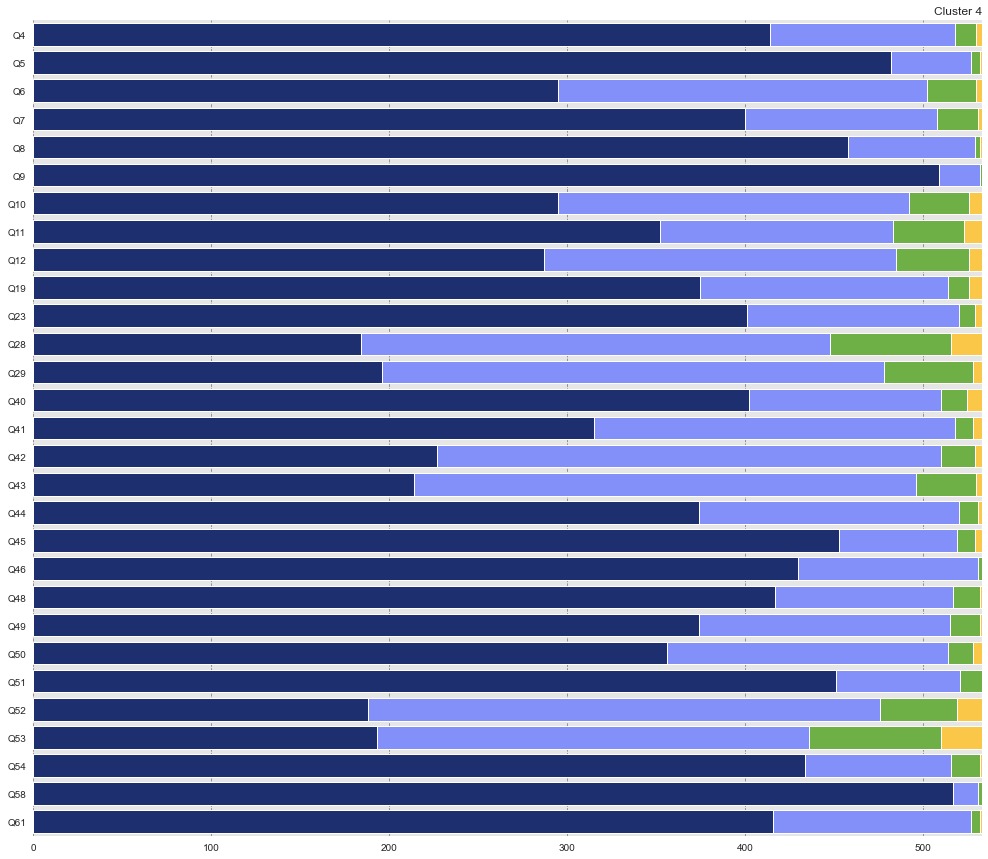


Supplementary Figure 3. Distribution of ordinal GI symptoms in overlapped clusters. The full description of variables is represented in Table 2.

Supplementary Figure 4. (Continued).


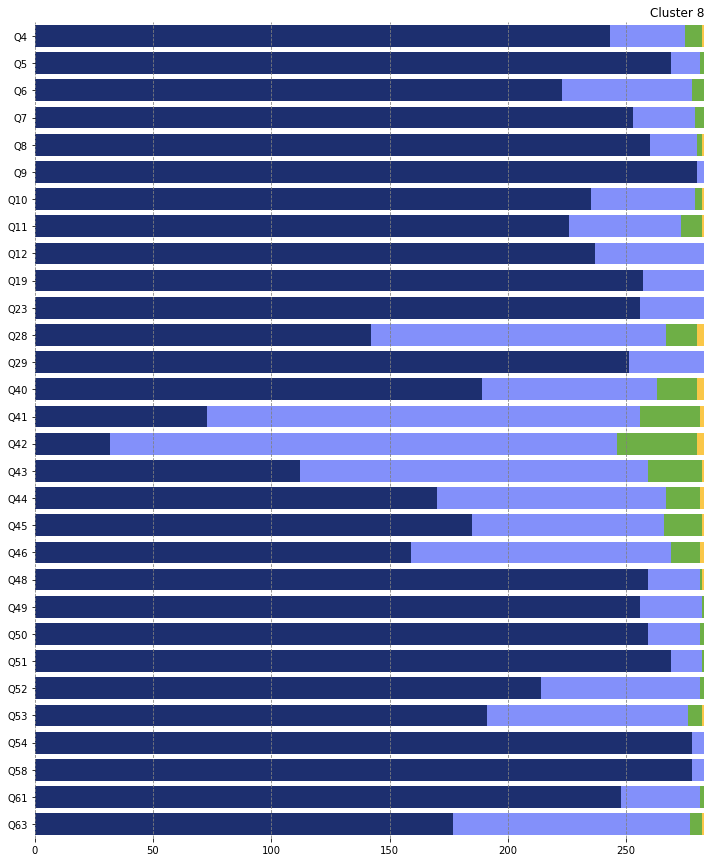

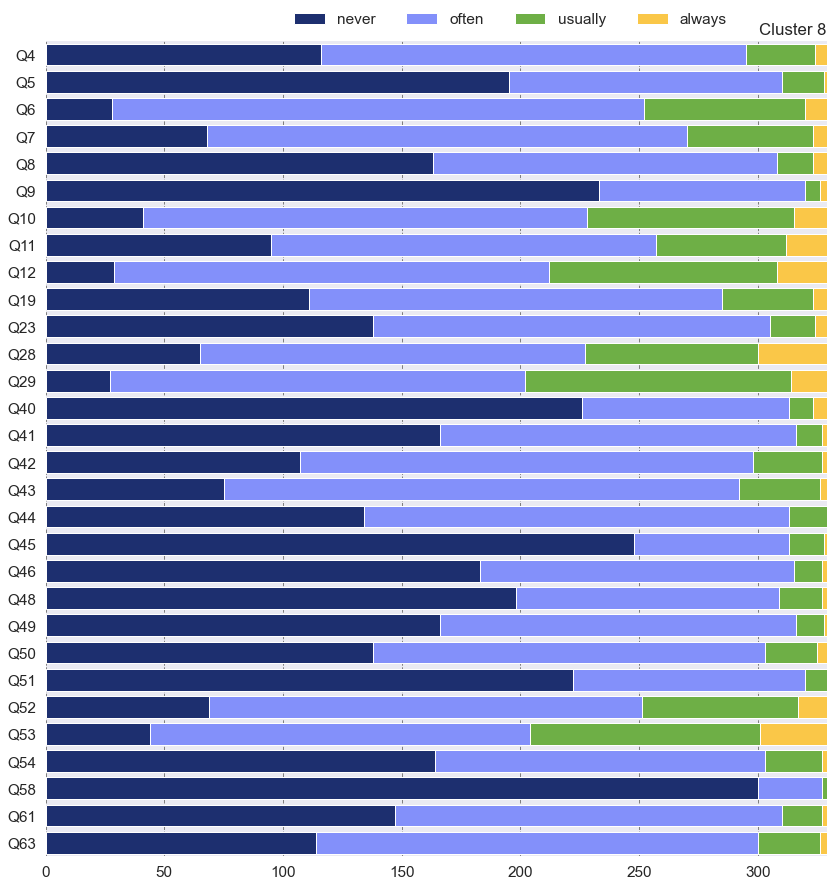

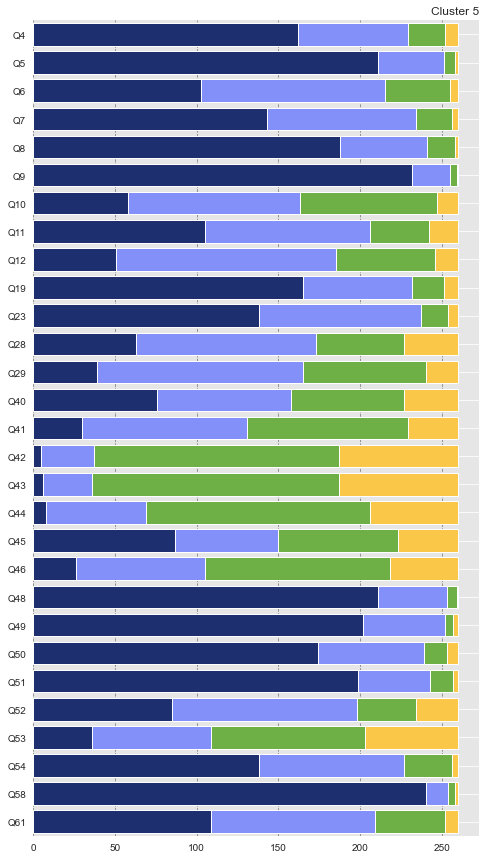

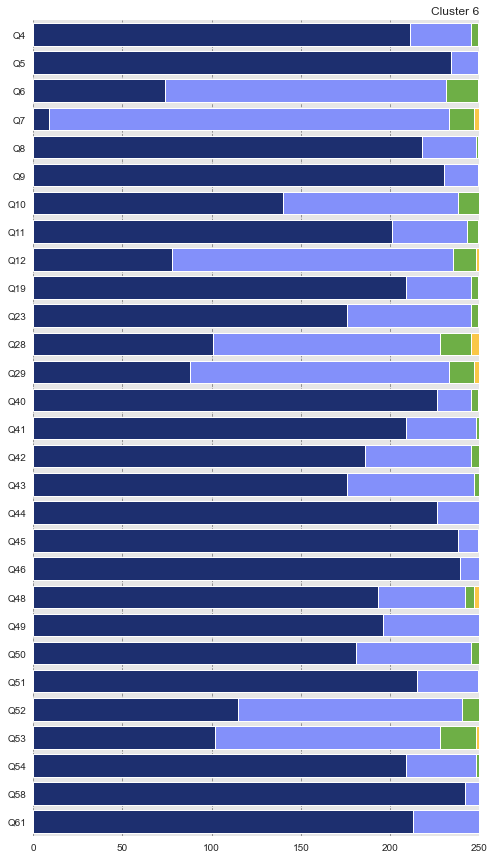

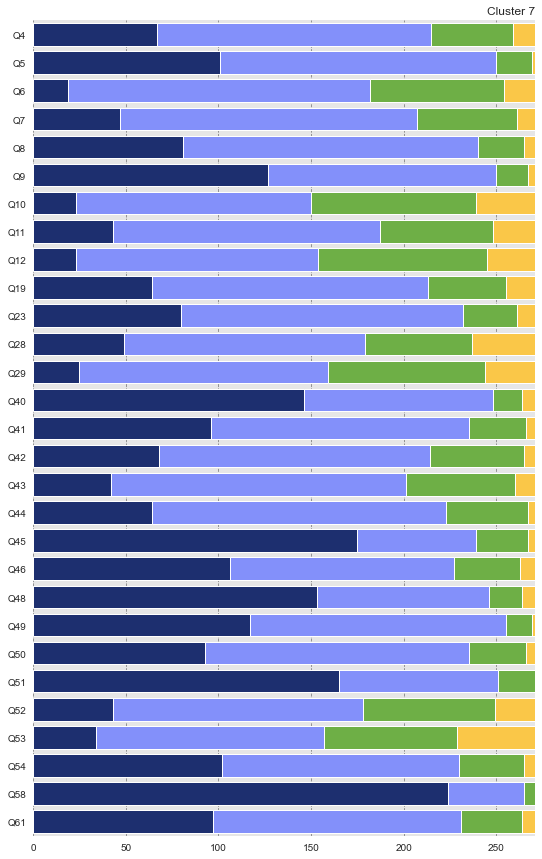


*
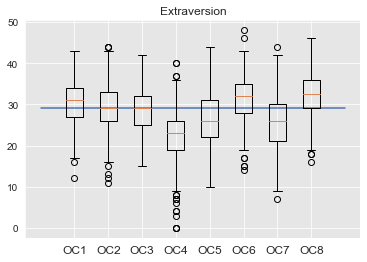

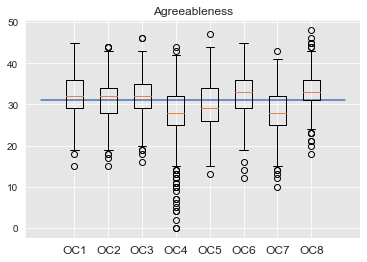

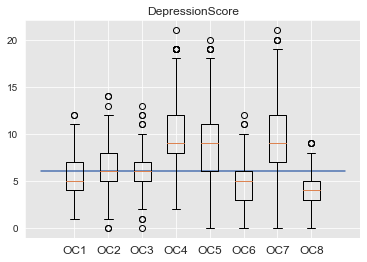

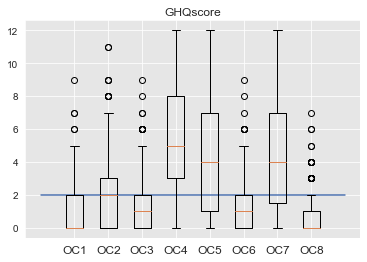

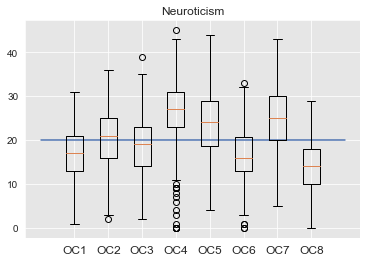

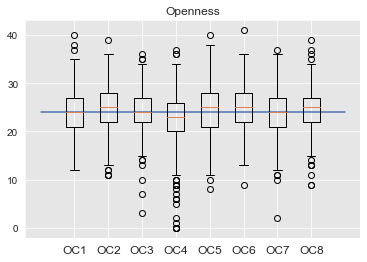

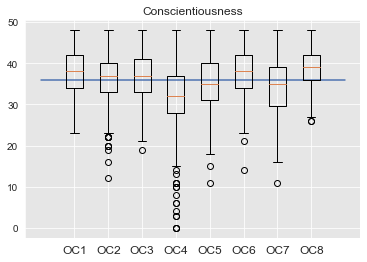

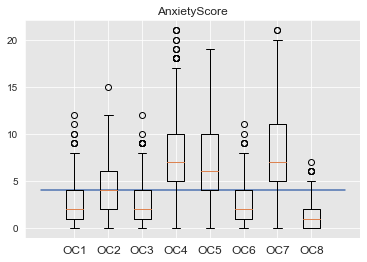
*

Supplementary Figure 5. Box plots of psychological factors in identified overlapped clusters (OC). The blue lines indicate the median of factors.


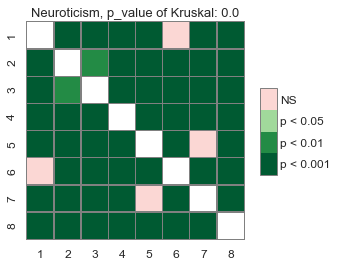

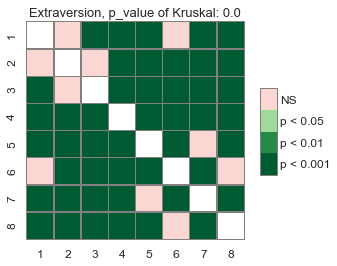

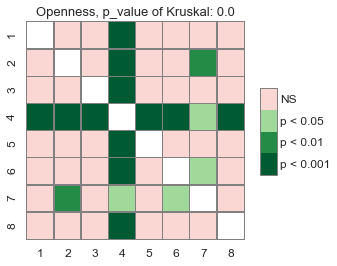

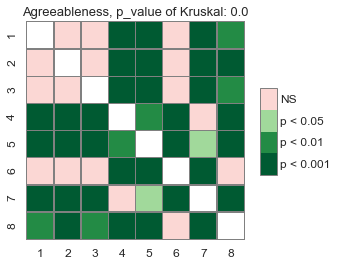

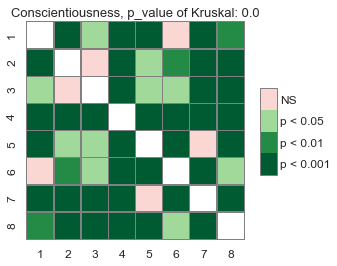

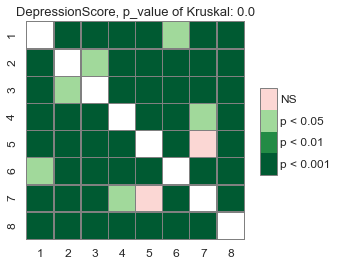

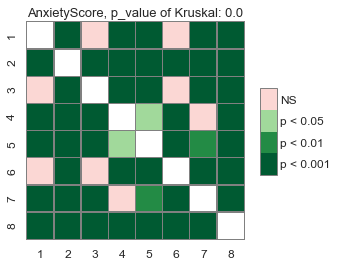

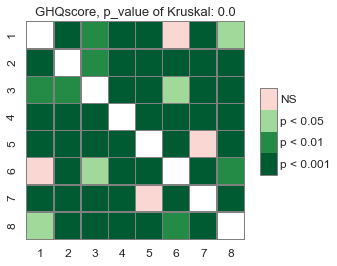

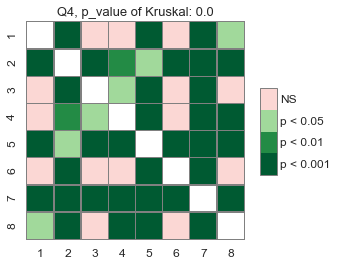

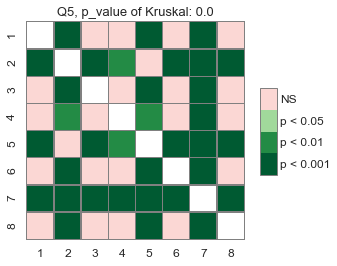

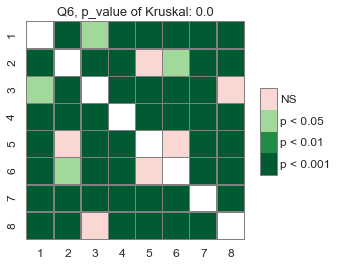

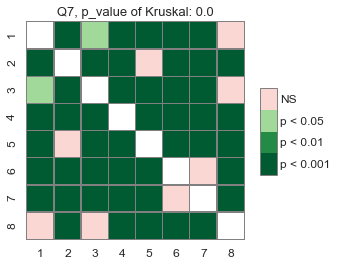

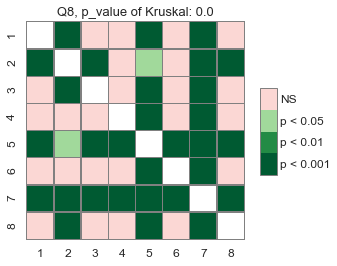

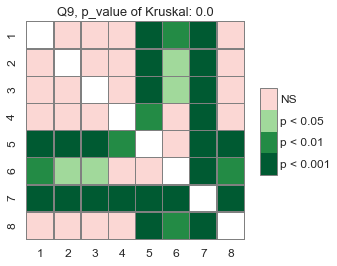

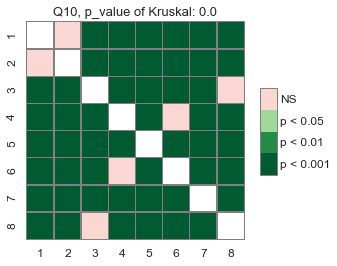

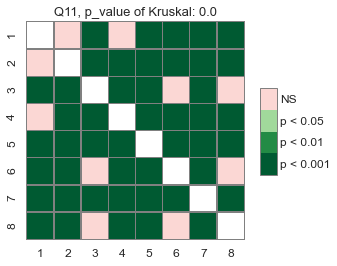

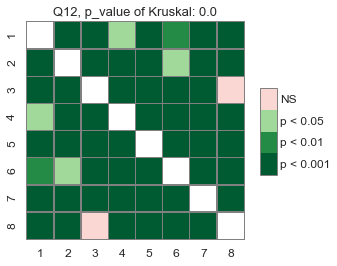

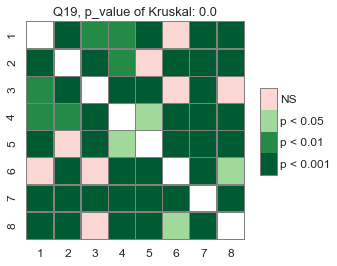

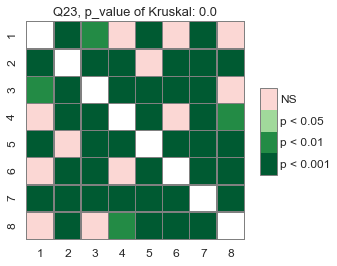

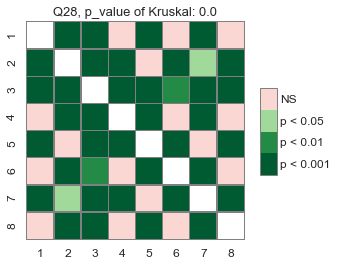


Supplementary Figure 6. Pairwise comparison of psychological factors, GI and somatic symptoms in 8 OCs, based on Game-Howell and Conover correction.


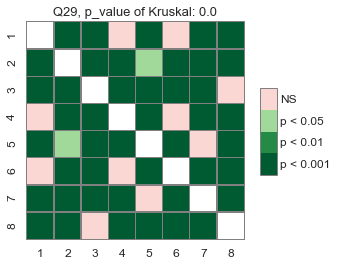

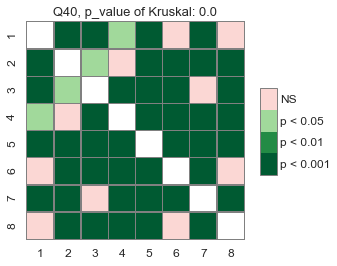

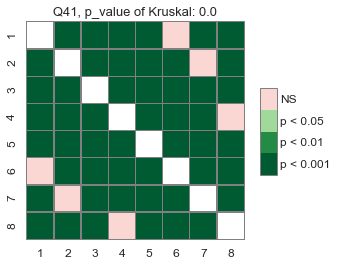

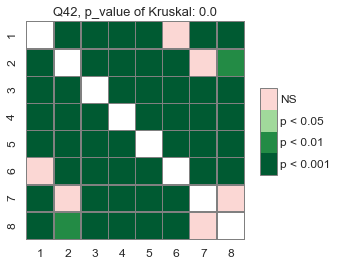

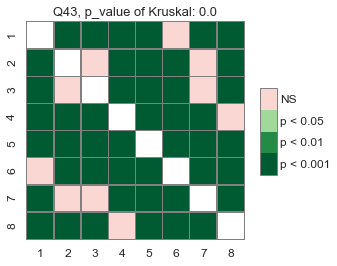

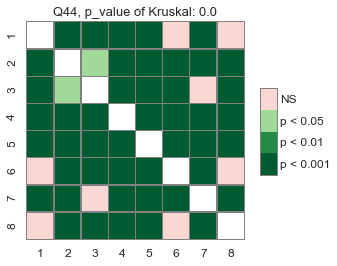

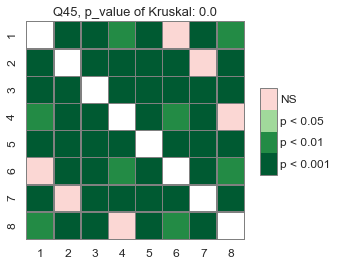

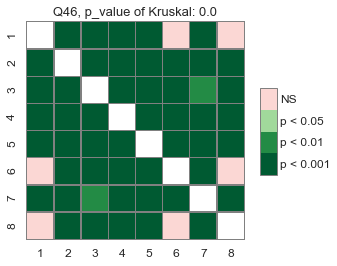

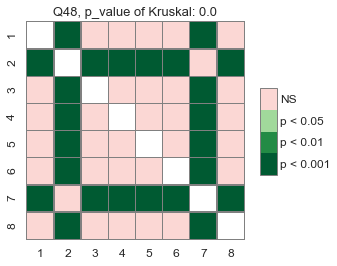

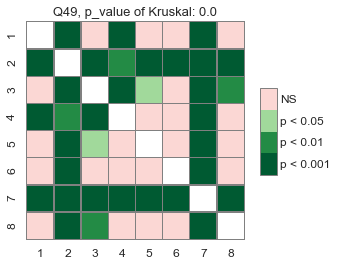

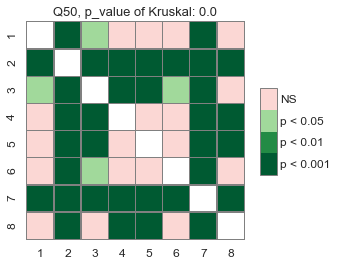

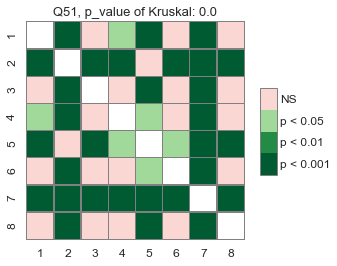

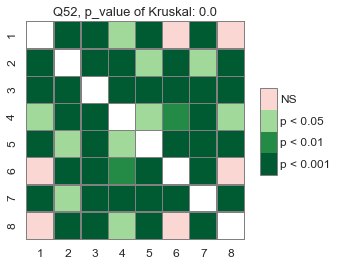

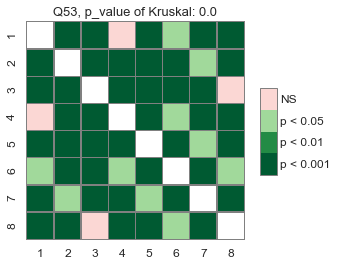

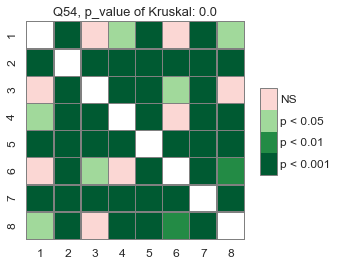

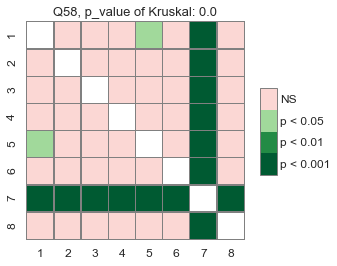

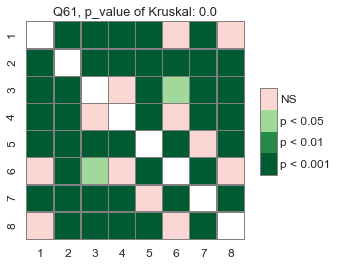

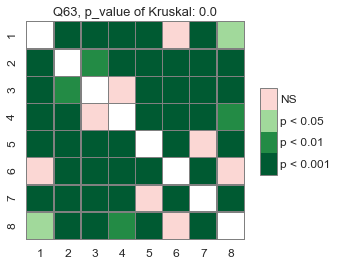


Supplementary Figure 7. (Continued)


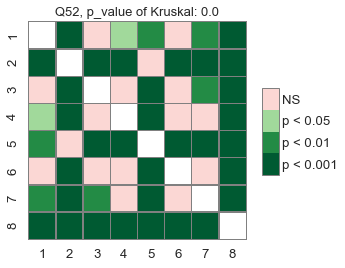

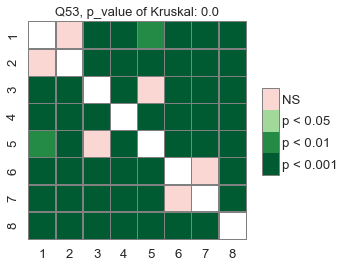

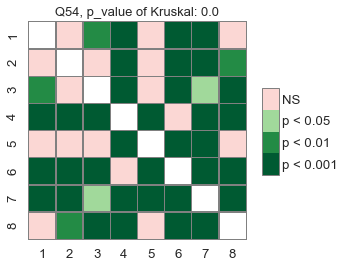

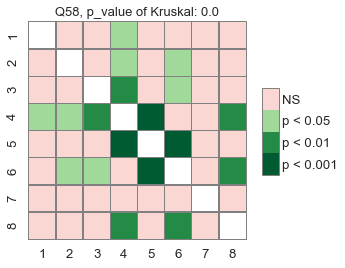

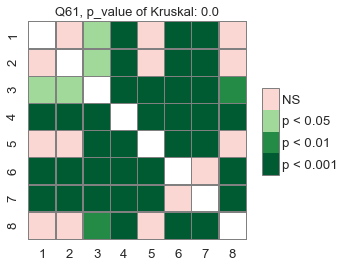

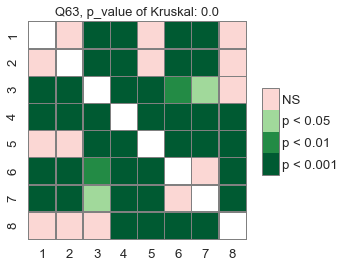


Supplementary Figure 8. (Continued), Q77: Headache, Q78: Backache, Q79: Asthma, Q80: Shortness of breath, Q81: Insomnia, Q82: Feeling exhausted, Q83: Stiffness, Q84: Heart palpitation, Q85: Joint pain, Q86: Eye pain, Q87: Dizziness, Q88: Feeling shivering, Q89: Flushing, Q90: High blood pressure.


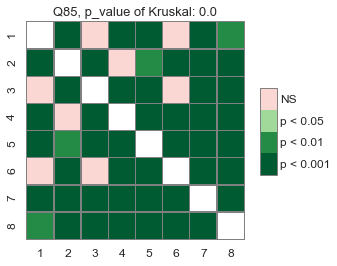

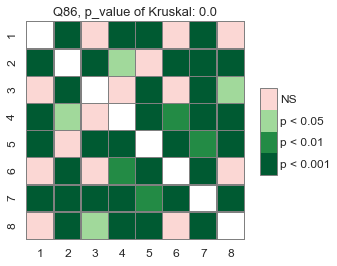

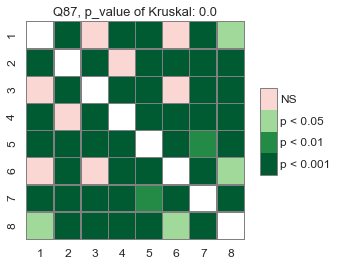

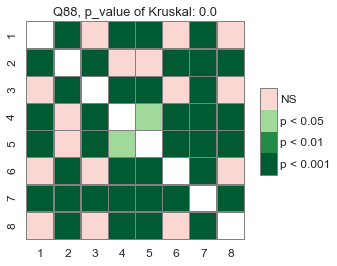

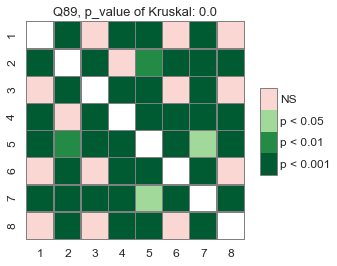

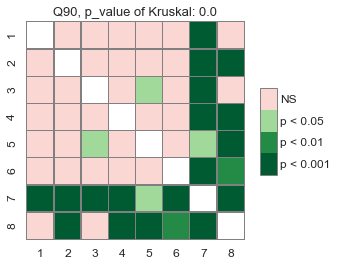

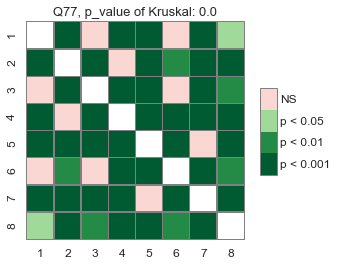

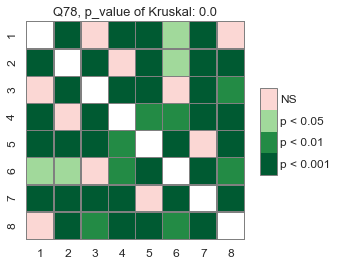

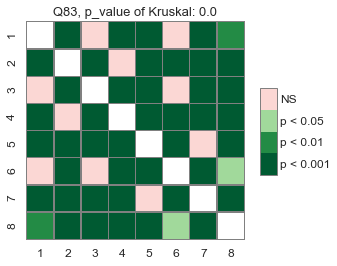

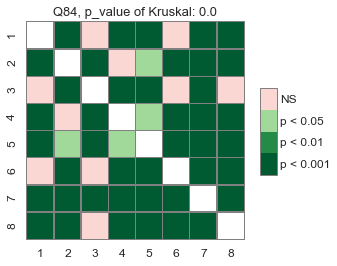


Supplementary Figure 9. Tracking the clustering procedure of step 2 in the following of non-pure clusters found in the first step.


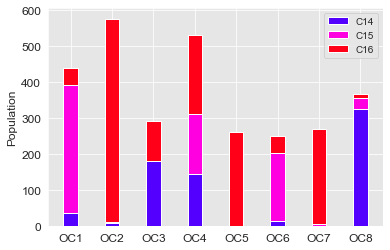


**Supplementary Tables**

Supplementary Table 1. Cross tabulation of ROME III disorders vs. first-step clustering results. The number of FGID patients in each cluster has been listed, and in parenthesis the percentage of cluster members with each FGID has been indicated. FHB indicates functional heartburn; FCP, functional chest pain; G, Globus; FDG, functional dysphagia; FDP, functional dyspepsia; PF, postprandial fullness; ES, epigastric pain syndrome; B, belching; V, Vomiting; FC, functional constipation; FB, functional bloating; FDI, functional diarrhea; FFI, functional fecal incontinence; FDF, functional defecation disorder; IBS, irritable bowel syndrome; IBS-C, constipation predominant IBS; IBS-D, diarrhea predominant IBS; IBS-M, mixed-type IBS; and IBS-U, unclassified IBS.

|  | **C1** | **C2** | **C3** | **C4** | **C5** | **C6** | **C7** | **C8** | **C9** | **C10** | **C11** | **C12** | **C13** | **C14** | **C15** | **C16** |
| --- | --- | --- | --- | --- | --- | --- | --- | --- | --- | --- | --- | --- | --- | --- | --- | --- |
| **Mean Age** | 32.2 | 34.3 | 23.9 | 38.0 | 24.4 | 32.2 | 30.7 | 29.0 | 29.6 | 32.9 | 35.2 | 33.6 | 31.3 | 32.5 | 31.5 | 31.8 |
| **Mean BMI** | 24.8 | 25.5 | 24 | 24.7 | 22.7 | 25.3 | 25.3 | 24.9 | 24.4 | 25.2 | 25.4 | 24.5 | 24.6 | 24.7 | 25.0 | 25.2 |
| **Female** | 8 | 27 | 10 | 13 | 9 | 61 | 25 | 28 | 27 | 41 | 45 | 43 | 14 | 398 | 405 | 1011 |
| **HB** | **25** | 21 | 0 | 1 | 7 | 0 | 0 | 2 | 1 | 4 | 4 | 2 | 1 | 64 | 242 | 704 |
| **(%)** | **(100)** | (29) | (0) | (4) | (19) | (0) | (0) | (5) | (2) | (4) | (6) | (3) | (3) | (9) | (32) | (46) |
| **FCP** | 0 | **51** | 1 | 11 | 1 | 3 | 5 | 5 | 0 | 7 | 3 | 0 | 24 | 125 | 196 | 385 |
| **(%)** |  | **(71)** | (4) | (39) | (3) | (2) | (12) | (11) |  | (7) | (4) |  | (80) | (18) | (26) | (25) |
| **G** | 0 | 0 | **23** | 0 | 2 | 0 | 0 | 2 | 1 | 6 | 1 | 5 | 0 | 52 | 67 | 128 |
| **(%)** |  |  | **(92)** |  | (6) |  |  | (5) | (2) | (6) | (1) | (7) |  | (7) | (9) | (8) |
| **FDG** | 0 | 0 | 2 | **25** | 1 | 0 | 1 | 2 | 0 | 2 | 1 | 0 | 0 | 41 | 49 | 185 |
| **(%)** |  |  | (8) | **(89)** | (3) |  | (2) | (5) |  | (2) | (1) |  |  | (6) | (7) | (12) |
| **FDP** | 0 | 0 | 0 | 0 | **17** | 0 | 0 | 0 | 0 | 0 | 0 | 0 | 2 | 18 | 112 | 551 |
| **(%)** |  |  | 0 | 0 | **(47)** |  |  |  |  |  |  |  | (7) | (3) | (15) | (36) |
| **PF** | 0 | 0 | 0 | 0 | **17** | 0 | 0 | 0 | 0 | 0 | 0 | 0 | 0 | 18 | 79 | 415 |
| **(%)** |  |  |  |  | **(47)** |  |  |  |  |  |  |  |  | (3) | (11) | (27) |
| **B** | 10 | 15 | 9 | 8 | 18 | **170** | **40** | 6 | 28 | 37 | 23 | 57 | 12 | 425 | 478 | 1152 |
| **(%)** | (40) | (21) | (36) | (29) | (50) | **(99)** | **(95)** | (14) | (44) | (39) | (33) | (78) | (40) | (60) | (64) | (75) |
| **V** | 0 | 2 | 0 | 1 | 3 | 6 | 0 | **44** | 2 | 2 | 3 | 9 | 1 | 74 | 168 | 668 |
| **(%)** |  | (3) |  | (4) | (8) | (3) |  | **(100)** | (3) | (2) | (4) | (12) | (3) | (10) | (22) | (44) |
| **FC** | 0 | 0 | 0 | 0 | 0 | 0 | 2 | 0 | 0 | 0 | **66** | 0 | 0 | 206 | 16 | 416 |
| **(%)** |  |  |  |  |  |  | (5) |  |  |  | **(96)** |  |  | (29) | (2) | (27) |
| **FB** | 1 | 2 | 0 | 0 | 1 | 0 | 7 | 3 | **63** | **96** | 2 | 28 | 1 | 204 | 315 | 182 |
| **(%)** | (4) | (3) |  |  | (3) |  | (17) | (7) | **(100)** | **(100)** | (3) | (38) | (3) | (29) | (42) | (12) |
| **IBS** | 0 | 0 | 0 | 0 | 0 | 0 | 0 | 2 | 0 | 0 | 0 | 14 | 5 | 57 | 155 | 755 |
| **(%)** |  |  |  |  |  |  |  | (5) |  |  |  | (19) | (17) | (8) | (21) | (49) |
| **IBS-C** | 0 | 0 | 0 | 0 | 0 | 0 | 0 | 0 | 0 | 0 | 0 | 0 | 0 | 30 | 12 | 292 |
| **(%)** |  |  |  |  |  |  |  |  |  |  |  |  |  | (4) | (2) | (19) |
| **IBS-D** | 0 | 0 | 0 | 0 | 0 | 0 | 0 | 0 | 0 | 0 | 0 | 4 | 0 | 3 | 59 | 136 |
| **(%)** |  |  |  |  |  |  |  |  |  |  |  | (5) |  |  | (8) | (9) |
| **IBS-M** | 0 | 0 | 0 | 0 | 0 | 0 | 0 | 0 | 0 | 0 | 0 | 1 | 0 | 5 | 4 | 175 |
| **(%)** |  |  |  |  |  |  |  |  |  |  |  | (1) |  | (1) | (1) | (11) |
| **IBS-U** | 0 | 0 | 0 | 0 | 0 | 0 | 0 | 2 | 0 | 0 | 0 | 9 | 5 | 19 | 80 | 152 |
| **(%)** |  |  |  |  |  |  |  | (5) |  |  |  | (12) | (17) | (3) | (11) | (10) |
| **FDI** | 0 | 0 | 0 | 0 | 0 | 0 | 0 | 0 | 0 | 1 | 0 | 0 | 0 | 2 | 5 | 7 |
| **(%)** |  |  |  |  |  |  |  |  |  | (1) |  |  |  |  | (1) |  |
| **FFI** | 0 | 0 | 0 | 1 | 0 | 0 | 0 | 0 | 0 | 0 | 1 | 0 | 0 | 16 | 13 | 116 |
| **(%)** |  |  |  | (4) |  |  |  |  |  |  | (1) |  |  | (2) | (2) | (8) |
| **FDF** | 0 | 0 | 0 | 0 | 0 | 0 | 0 | 0 | 0 | 0 | 2 | 0 | 0 | 49 | 2 | 431 |
| **(%)** |  |  |  |  |  |  |  |  |  |  | (3) |  |  | (7) |  | (28) |
| **COM.** | 0.12 | 0.15 | 0.16 | 0.26 | 0.31 | 0.09 | 0.18 | 0.25 | 0.13 | 0.22 | 0.27 | 0.22 | 0.19 | 0.62 | 0.67 | 1.23 |
| **No. of SAMPLES** | 25 | 72 | 25 | 28 | 36 | 172 | 42 | 44 | 63 | 96 | 69 | 73 | 30 | 707 | 752 | 1531 |

Supplementary Table 2. Results of Multiple comparison test (MCT) of 8 identified OCs based on Kruskal-Wallis and the Man-Whitney U test for comparison of each OC vs. the rest of population.

|  | OC1 vs. others | | OC2 vs. others | | OC3 vs. others | | OC4 vs. others | | OC5 vs. others | | OC6 vs. others | | OC7 vs. others | | OC8 vs. others | | MCT | |
| --- | --- | --- | --- | --- | --- | --- | --- | --- | --- | --- | --- | --- | --- | --- | --- | --- | --- | --- |
| Variables | p-value | Effect  Size | p-value | Effect  Size | p-value | Effect  Size | p-value | Effect  Size | p-value | Effect  Size | p-value | Effect  Size | p-value | Effect  Size | p-value | Effect  Size | p-value | Effect  Size |
| Neuro. | 0.0 | 0.32 | 0.863 | 0.0 | 0.0 | 0.13 | 0.0 | 0.57 | 0.0 | 0.28 | 0.0 | 0.33 | 0.0 | 0.35 | 0.0 | 0.55 | 0.0 | 0.31 |
| Extra. | 0.0 | 0.22 | 0.0 | 0.13 | 0.215 | 0.04 | 0.0 | 0.6 | 0.0 | 0.18 | 0.0 | 0.32 | 0.0 | 0.23 | 0.0 | 0.41 | 0.0 | 0.24 |
| Open. | 0.637 | 0.01 | 0.0 | 0.12 | 0.828 | 0.01 | 0.0 | 0.23 | 0.55 | 0.02 | 0.006 | 0.1 | 0.09 | 0.06 | 0.038 | 0.07 | 0.0 | 0.03 |
| Agree. | 0.0 | 0.15 | 0.013 | 0.07 | 0.001 | 0.12 | 0.0 | 0.34 | 0.0 | 0.14 | 0.0 | 0.17 | 0.0 | 0.29 | 0.0 | 0.3 | 0.0 | 0.11 |
| Cons. | 0.0 | 0.19 | 0.29 | 0.03 | 0.213 | 0.04 | 0.0 | 0.42 | 0.009 | 0.1 | 0.0 | 0.18 | 0.0 | 0.15 | 0.0 | 0.34 | 0.0 | 0.12 |
| Dep. | 0.0 | 0.31 | 0.034 | 0.06 | 0.0 | 0.14 | 0.0 | 0.61 | 0.0 | 0.37 | 0.0 | 0.39 | 0.0 | 0.46 | 0.0 | 0.61 | 0.0 | 0.39 |
| AnX. | 0.0 | 0.35 | 0.538 | 0.02 | 0.0 | 0.28 | 0.0 | 0.55 | 0.0 | 0.4 | 0.0 | 0.35 | 0.0 | 0.55 | 0.0 | 0.61 | 0.0 | 0.41 |
| GHQ | 0.0 | 0.36 | 0.001 | 0.09 | 0.0 | 0.19 | 0.0 | 0.63 | 0.0 | 0.33 | 0.0 | 0.3 | 0.0 | 0.36 | 0.0 | 0.46 | 0.0 | 0.34 |
| Q4 | 0.0 | 0.12 | 0.01 | 0.05 | 0.0 | 0.15 | 0.016 | 0.05 | 0.0 | 0.14 | 0.0 | 0.12 | 0.0 | 0.56 | 0.0 | 0.2 | 0.0 | 0.17 |
| Q5 | 0.0 | 0.09 | 0.137 | 0.02 | 0.0 | 0.11 | 0.0 | 0.07 | 0.051 | 0.05 | 0.0 | 0.09 | 0.0 | 0.53 | 0.0 | 0.12 | 0.0 | 0.2 |
| Q6 | 0.0 | 0.21 | 0.0 | 0.13 | 0.0 | 0.3 | 0.019 | 0.06 | 0.0 | 0.17 | 0.0 | 0.22 | 0.0 | 0.58 | 0.0 | 0.35 | 0.0 | 0.21 |
| Q7 | 0.0 | 0.37 | 0.0 | 0.08 | 0.0 | 0.26 | 0.0 | 0.11 | 0.0 | 0.13 | 0.0 | 0.65 | 0.0 | 0.57 | 0.0 | 0.33 | 0.0 | 0.38 |
| Q8 | 0.0 | 0.12 | 0.836 | 0.0 | 0.0 | 0.12 | 0.0 | 0.07 | 0.0 | 0.1 | 0.005 | 0.07 | 0.0 | 0.56 | 0.0 | 0.14 | 0.0 | 0.19 |
| Q9 | 0.0 | 0.08 | 0.0 | 0.07 | 0.0 | 0.07 | 0.001 | 0.04 | 0.102 | 0.03 | 0.9 | 0.0 | 0.0 | 0.5 | 0.0 | 0.09 | 0.0 | 0.28 |
| Q10 | 0.0 | 0.12 | 0.0 | **0.19** | 0.0 | 0.41 | 0.0 | 0.13 | 0.0 | 0.38 | 0.0 | 0.14 | 0.0 | 0.56 | 0.0 | 0.51 | 0.0 | 0.29 |
| Q11 | 0.415 | 0.02 | 0.001 | 0.08 | 0.0 | 0.24 | 0.016 | 0.06 | 0.0 | 0.25 | 0.0 | 0.22 | 0.0 | 0.54 | 0.0 | 0.3 | 0.0 | 0.18 |
| Q12 | 0.296 | 0.03 | 0.0 | **0.21** | 0.0 | 0.45 | 0.0 | 0.11 | 0.0 | 0.35 | 0.004 | 0.1 | 0.0 | 0.55 | 0.0 | 0.5 | 0.0 | 0.29 |
| Q19 | 0.0 | 0.11 | 0.0 | 0.11 | 0.0 | 0.23 | 0.812 | 0.01 | 0.001 | 0.1 | 0.0 | 0.14 | 0.0 | 0.55 | 0.0 | 0.25 | 0.0 | 0.18 |
| Q23 | 0.0 | 0.13 | 0.0 | 0.14 | 0.0 | 0.26 | 0.0 | 0.08 | 0.0 | 0.18 | 0.437 | 0.02 | 0.0 | 0.45 | 0.0 | 0.19 | 0.0 | 0.15 |
| Q28 | 0.01 | 0.07 | 0.0 | 0.15 | 0.0 | 0.33 | 0.07 | 0.05 | 0.0 | 0.19 | 0.0 | 0.15 | 0.0 | 0.25 | 0.244 | 0.03 | 0.0 | 0.08 |
| Q29 | 0.0 | 0.11 | 0.0 | 0.3 | 0.0 | 0.44 | 0.307 | 0.03 | 0.0 | 0.37 | 0.368 | 0.03 | 0.0 | 0.46 | 0.0 | 0.51 | 0.0 | 0.28 |
| Q40 | 0.0 | 0.16 | 0.557 | 0.01 | 0.0 | 0.11 | 0.003 | 0.07 | 0.0 | 0.52 | 0.0 | 0.22 | 0.0 | 0.17 | 0.0 | 0.19 | 0.0 | 0.16 |
| Q41 | 0.0 | 0.44 | 0.0 | 0.11 | 0.0 | 0.34 | 0.0 | 0.13 | 0.0 | 0.62 | 0.0 | 0.38 | 0.0 | 0.17 | 0.036 | 0.06 | 0.0 | 0.28 |
| Q42 | 0.0 | 0.57 | 0.0 | 0.17 | 0.0 | 0.29 | 0.0 | 0.18 | 0.0 | 0.83 | 0.0 | 0.47 | 0.0 | 0.12 | 0.056 | 0.06 | 0.0 | 0.42 |
| Q43 | 0.0 | 0.44 | 0.0 | 0.23 | 0.0 | 0.15 | 0.0 | 0.15 | 0.0 | 0.82 | 0.0 | 0.45 | 0.0 | 0.23 | 0.0 | **0.2** | 0.0 | 0.37 |
| Q44 | 0.0 | 0.43 | 0.0 | 0.24 | 0.0 | 0.3 | 0.0 | 0.21 | 0.0 | 0.82 | 0.0 | 0.4 | 0.0 | 0.34 | 0.0 | **0.38** | 0.0 | 0.46 |
| Q45 | 0.0 | 0.24 | 0.0 | 0.07 | 0.0 | 0.25 | 0.0 | 0.14 | 0.0 | 0.51 | 0.0 | 0.24 | 0.0 | 0.11 | 0.0 | **0.13** | 0.0 | 0.2 |
| Q46 | 0.0 | 0.38 | 0.0 | 0.17 | 0.0 | 0.41 | 0.0 | 0.23 | 0.0 | 0.75 | 0.0 | 0.36 | 0.0 | 0.28 | 0.0 | **0.33** | 0.0 | 0.43 |
| Q48 | 0.007 | 0.06 | 0.0 | 0.14 | 0.0 | 0.13 | 0.054 | 0.04 | 0.017 | 0.07 | 0.401 | 0.02 | 0.0 | 0.21 | 0.014 | 0.06 | 0.0 | 0.04 |
| Q49 | 0.0 | 0.13 | 0.0 | 0.13 | 0.0 | 0.19 | 0.425 | 0.02 | 0.025 | 0.07 | 0.006 | 0.08 | 0.0 | 0.31 | 0.105 | 0.04 | 0.0 | 0.07 |
| Q50 | 0.0 | 0.11 | 0.0 | 0.2 | 0.0 | 0.21 | 0.4 | 0.02 | 0.97 | 0.0 | 0.009 | 0.08 | 0.0 | 0.36 | 0.0 | 0.18 | 0.0 | 0.1 |
| Q51 | 0.0 | 0.11 | 0.0 | 0.1 | 0.0 | 0.09 | 0.085 | 0.03 | 0.008 | 0.07 | 0.066 | 0.05 | 0.0 | 0.24 | 0.0 | 0.09 | 0.0 | 0.06 |
| Q52 | 0.0 | 0.13 | 0.0 | 0.26 | 0.0 | 0.38 | 0.628 | 0.01 | 0.001 | 0.11 | 0.0 | 0.16 | 0.0 | 0.35 | 0.0 | 0.13 | 0.0 | 0.13 |
| Q53 | 0.024 | 0.06 | 0.0 | 0.25 | 0.0 | 0.37 | 0.002 | 0.08 | 0.0 | 0.44 | 0.0 | 0.18 | 0.0 | 0.33 | 0.0 | 0.33 | 0.0 | 0.19 |
| Q54 | 0.0 | 0.14 | 0.0 | 0.1 | 0.0 | 0.19 | 0.003 | 0.06 | 0.0 | 0.27 | 0.003 | 0.08 | 0.0 | 0.44 | 0.0 | 0.22 | 0.0 | 0.17 |
| Q58 | 0.006 | 0.03 | 0.656 | 0.0 | 0.039 | 0.03 | 0.029 | 0.02 | 0.022 | 0.03 | 0.2 | 0.02 | 0.0 | 0.14 | 0.077 | 0.02 | 0.0 | 0.04 |
| Q61 | 0.0 | 0.24 | 0.0 | 0.2 | 0.025 | 0.06 | 0.0 | 0.11 | 0.0 | 0.34 | 0.0 | 0.18 | 0.0 | 0.39 | 0.0 | 0.24 | 0.0 | 0.19 |
| Q63 | 0.0 | 0.31 | 0.0 | 0.16 | 0.558 | 0.02 | 0.028 | 0.05 | 0.0 | 0.37 | 0.0 | 0.21 | 0.0 | 0.32 | 0.0 | 0.19 | 0.0 | 0.15 |
| HeadAche | 0.0 | 0.15 | 0.035 | 0.05 | 0.0 | 0.12 | 0.0 | 0.1 | 0.0 | **0.25** | 0.003 | 0.1 | 0.0 | **0.29** | 0.0 | **0.27** | 0.0 | 0.09 |
| backache | 0.0 | 0.21 | 0.01 | 0.06 | 0.0 | 0.12 | 0.0 | 0.09 | 0.0 | **0.24** | 0.03 | 0.08 | 0.0 | **0.35** | 0.0 | **0.26** | 0.0 | 0.1 |
| Asthma | 0.0 | 0.08 | 0.028 | 0.04 | 0.041 | 0.04 | 0.619 | 0.01 | 0.0 | 0.09 | 0.023 | 0.05 | 0.0 | **0.22** | 0.0 | 0.1 | 0.0 | 0.05 |
| Shortness of breath | 0.0 | 0.17 | 0.0 | 0.11 | 0.0 | 0.12 | 0.005 | 0.07 | 0.0 | 0.2 | 0.0 | 0.18 | 0.0 | **0.42** | 0.0 | **0.3** | 0.0 | 0.13 |
| insomnia | 0.0 | 0.17 | 0.004 | 0.07 | 0.011 | 0.08 | 0.0 | 0.13 | 0.0 | 0.15 | 0.0 | 0.18 | 0.0 | **0.34** | 0.0 | **0.25** | 0.0 | 0.09 |
| feeling exhausted | 0.0 | **0.22** | 0.207 | 0.03 | 0.0 | 0.14 | 0.0 | **0.23** | 0.0 | **0.32** | 0.0 | **0.26** | 0.0 | **0.41** | 0.0 | **0.35** | 0.0 | 0.18 |
| STIFFNESS | 0.0 | 0.18 | 0.0 | 0.1 | 0.004 | 0.09 | 0.004 | 0.07 | 0.0 | **0.25** | 0.0 | 0.17 | 0.0 | **0.36** | 0.0 | **0.31** | 0.0 | 0.11 |
| heart PALPITATION | 0.0 | 0.19 | 0.0 | 0.11 | 0.0 | 0.23 | 0.0 | 0.11 | 0.0 | **0.21** | 0.0 | 0.14 | 0.0 | **0.48** | 0.0 | **0.33** | 0.0 | 0.16 |
| joint pain | 0.0 | **0.21** | 0.0 | 0.11 | 0.001 | 0.11 | 0.029 | 0.06 | 0.0 | **0.24** | 0.002 | 0.11 | 0.0 | **0.44** | 0.0 | **0.34** | 0.0 | 0.13 |
| Eye pain | 0.0 | 0.16 | 0.0 | 0.11 | 0.013 | 0.08 | 0.983 | 0.0 | 0.0 | 0.18 | 0.0 | 0.15 | 0.0 | **0.36** | 0.0 | **0.21** | 0.0 | 0.08 |
| Dizziness | 0.0 | **0.23** | 0.0 | 0.1 | 0.0 | 0.14 | 0.0 | 0.13 | 0.0 | 0.28 | 0.0 | 0.2 | 0.0 | **0.44** | 0.0 | **0.33** | 0.0 | 0.16 |
| Feeling shivering | 0.0 | 0.17 | 0.0 | 0.1 | 0.0 | 0.14 | 0.013 | 0.06 | 0.0 | 0.16 | 0.0 | 0.15 | 0.0 | **0.4** | 0.0 | **0.23** | 0.0 | 0.11 |
| FLUSHING | 0.0 | 0.16 | 0.0 | 0.09 | 0.0 | 0.14 | 0.01 | 0.06 | 0.0 | **0.23** | 0.0 | 0.16 | 0.0 | **0.37** | 0.0 | **0.25** | 0.0 | 0.11 |
| High blood pressure | 0.039 | 0.03 | 0.288 | 0.02 | 0.004 | 0.06 | 0.646 | 0.01 | 0.039 | 0.04 | 0.713 | 0.01 | 0.0 | 0.14 | 0.0 | 0.09 | 0.0 | 0.03 |

Supplementary Table 3. Proportion of ROME Disorders in eight identified overlapped clusters (OCs).

| Disorders | OC1 | OC2 | OC3 | OC4 | OC5 | OC6 | OC7 | OC8 |
| --- | --- | --- | --- | --- | --- | --- | --- | --- |
| FHB | 10 | 235 | 32 | 133 | 117 | 241 | 224 | 18 |
| FCP | 142 | 178 | 53 | 154 | 63 | 9 | 39 | 68 |
| G | 55 | 65 | 20 | 54 | 23 | 1 | 4 | 25 |
| FDg | 40 | 71 | 23 | 52 | 29 | 0 | 33 | 27 |
| FDp | 80 | 129 | 14 | 103 | 142 | 30 | 178 | 5 |
| PF | 59 | 82 | 13 | 73 | 116 | 18 | 147 | 4 |
| B | 290 | 459 | 123 | 349 | 197 | 149 | 222 | 266 |
| V | 98 | 226 | 28 | 165 | 102 | 44 | 211 | 36 |
| FC | 25 | 126 | 188 | 95 | 89 | 17 | 43 | 55 |
| FB | 199 | 108 | 30 | 124 | 5 | 83 | 18 | 134 |
| IBS | 85 | 301 | 32 | 147 | 153 | 64 | 157 | 28 |
| IBS-C | 8 | 115 | 22 | 39 | 104 | 7 | 31 | 8 |
| IBS-D | 22 | 53 | 3 | 45 | 9 | 21 | 37 | 8 |
| IBS-M | 1 | 67 | 2 | 16 | 32 | 3 | 61 | 2 |
| IBS-U | 54 | 66 | 5 | 47 | 8 | 33 | 28 | 10 |
| FDi | 4 | 2 | 1 | 4 | 1 | 0 | 2 | 0 |
| FFI | 10 | 26 | 7 | 16 | 20 | 8 | 47 | 11 |
| FDf | 7 | 72 | 101 | 45 | 200 | 5 | 41 | 11 |
| No. of Samples | 441 | 577 | 292 | 533 | 260 | 250 | 271 | 366 |
